# Cage bedding modifies metabolic and gut microbiota profiles in mouse studies applying dietary restriction

**DOI:** 10.1038/s41598-020-77831-3

**Published:** 2020-11-30

**Authors:** A. Gregor, L. Fragner, S. Trajanoski, W. Li, X. Sun, W. Weckwerth, J. König, K. Duszka

**Affiliations:** 1 Department of Nutritional Sciences, University of Vienna, Althanstrasse 14 (UZA II), 1090 Wien, Austria; 2 Molecular Systems Biology, Department of Functional and Evolutionary Ecology, University of Vienna, Vienna, Austria; 3 Vienna Metabolomics Center, University of Vienna, Vienna, Austria; 4 Core Facility Computational Bioanalytics, Medical University of Graz, Graz, Austria

**Keywords:** Microbiome, Metabolomics, Molecular biology

## Abstract

Experiments involving food restriction are common practice in metabolic research. Under fasted conditions, mice supplement their diet with cage bedding. We aimed at identifying metabolic and microbiota-related parameters affected by the bedding type. We exposed mice housed with wooden, cellulose, or corncob cage beddings to ad libitum feeding, caloric restriction (CR), or over-night (ON) fasting. Additionally, two subgroups of the ON fast group were kept without any bedding or on a metal grid preventing coprophagy. Mice under CR supplemented their diet substantially with bedding; however, the amount varied depending on the kind of bedding. Bedding-related changes in body weight loss, fat loss, cecum size, stomach weight, fecal output, blood ghrelin levels as well as a response to glucose oral tolerance test were recorded. As fiber is fermented by the gut bacteria, the type of bedding affects gut bacteria and fecal metabolites composition of CR mice. CR wood and cellulose groups showed distinct cecal metabolite and microbiome profiles when compared to the CR corncob group. While all ad libitum fed animal groups share similar profiles. We show that restriction-related additional intake of bedding-derived fiber modulates multiple physiological parameters. Therefore, the previous rodent studies on CR, report the combined effect of CR and increased fiber consumption.

## Introduction

It is commonly agreed that mice need to be housed with bedding and nesting material to fulfill the animals’ welfare requirements. Cage bedding is an important factor for animal well-being. It provides nesting material, helps to keep warmth, provides a proper walking surface, and buffers air ammonia content^1^. However, mice and rats fed at ad libitum and, even more, under dietary restrictions tend to consume cage bedding^2^. Moreover, multiple metabolic tests involving rodents are preceded by over-night (ON) fasting. Thus, the type of bedding and mice’s ability to extract energy from the bedding will impact the results of the metabolic tests. An even bigger impact is expected in the case of caloric restriction (CR). CR requires daily delivery of a limited, accurately dosed amount of food. In animal as well as in human studies CR has been shown to increase lifespan and health-span. It prevents the development of various diseases including age-related, neurological, and metabolic diseases as well as cancer^3,4^. For a successful CR experimental protocol, mice energy intake has to be strictly adjusted. Thus, uncontrolled energy uptake and fiber supplementation by consuming cage bedding can substantially influence the experimental outcome. Accordingly, in our previous publication^5^, we showed that mice submitted to CR develop an increased cecum size. We concluded that the enlarged cecum results from the accumulation of indigestible fiber as a consequence of bedding consumption. The enlarged cecum is observed upon consumption of a high-fiber diet^6–8^ but it is also a phenotype typical to germ-free mice and indicates aggregation of fiber mass as well as disturbed nitrogen reabsorption in the small intestine^9–11^. Importantly, fiber can also reduce nutrient digestibility as it limits the access of enzymes to nutrients and it may also affect the passage rate of the digesta, especially if consumed in high amounts^12^. Accordingly, it has been noticed that feed conversion is reduced from animals housed on corncob (CC) bedding^13^. Two previous publications addressed the issue of the impact of the CC bedding on mice body weight and gut microbiota concluding that application of the CC bedding may confound study results^13,14^. However, no one, thus far, compared different types of bedding considering metabolic research and microbiota.

Bedding consumption is particularly important in the context of the rapidly developing field of gut microbiota. Dietary fibers, including those present in cage bedding, may undergo complete or partial fermentation by the gut microbes^15^, leading to the production of short-chain fatty acids (SCFA) and stimulating the growth of certain bacterial species^16^. The metabolic end products of colonic microbiota play an important role in the maintenance of health and the development of disease^15,17^. Succeeding inconsistencies in scientific outcomes, the issue of reproducibility in microbiota research was inevitably raised^18,19^. We aim at studying the variability in research outcomes introduced by cage beddings as well as bringing awareness to the reproducibility of the studies involving fasting and CR by stressing the importance of bedding.

## Results

### Cage bedding affects mice body and organ weight

**Figure 1:**
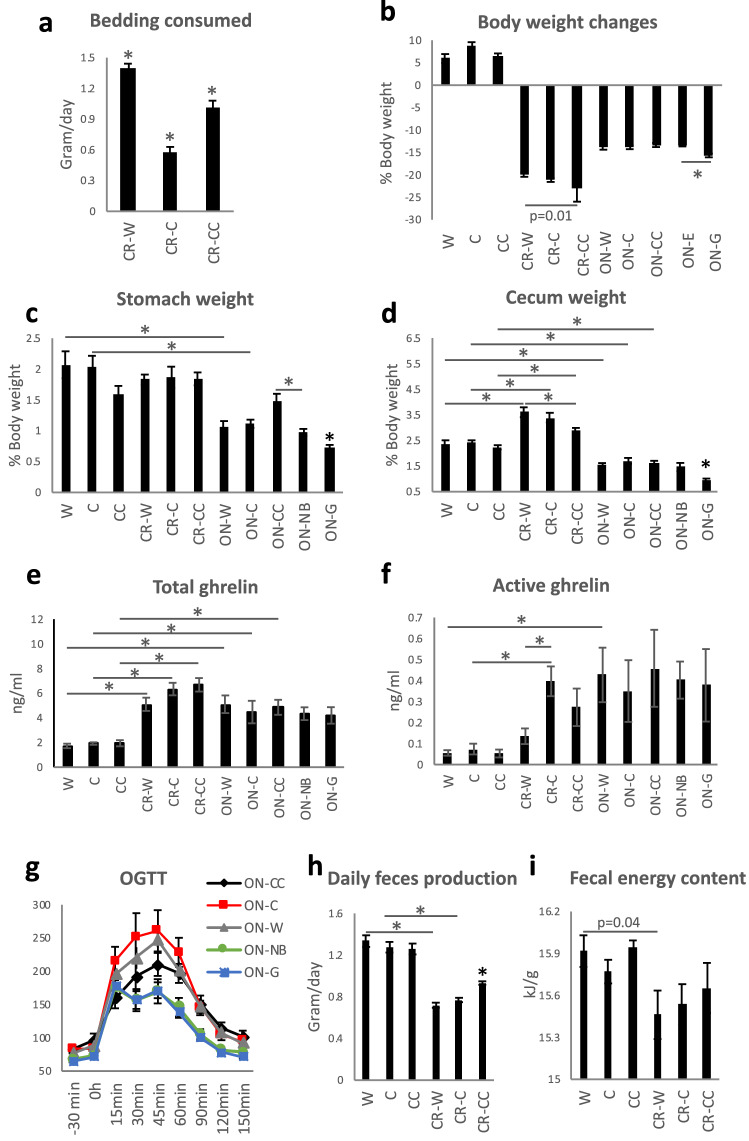
Cage bedding affects body parameters in dietary restricted mice. The amount of bedding consumed by mice submitted caloric restriction (CR) was measured daily between days 11 and 13 of the CR (**a**). Bodyweight changes were recorded for ad libitum, CR mice as well as over-night (ON) fasted mice and expressed as % change (**b**). Stomach (**c**) and cecum (**d**) weight were measured. Total (**e**) and active (**f**) ghrelin concentrations were analyzed in the mice plasma. Oral glucose tolerance test (OGTT) was performed after ON fasting (**g**). CR mice feces were collected and its weight (**h**), as well as energy content (**i**), was assessed. One-way ANOVA was applied to verify statistical significance. Asterisk (*) indicates statistical significance between the indicated groups after Bonferroni correction for multiple testing. The bars indicate the mean of eight to ten biological replicates ± SEM.

To assay the impact of cage bedding we submitted mice to two kinds of dietary restrictions in different housing conditions. The mice were held with one of the cage beddings: wooden (W), cellulose (C), or corncob (CC). The animals from over-night fasted (ON) groups were housed with one of three cage beddings (ON-W, ON-C, ON-CC) and additional groups without bedding (no bedding, ON-NB) or on a metal grid (ON-G). The grid was preventing the animals from contact with the cage bottom, thus limiting coprophagy. In parallel, to assess the long-term effects of bedding consumption we submitted mice to 14 days CR using the three kinds of cage bedding: wooden (CR-W), cellulose (CR-C), and corncob (CR-CC). Corresponding control groups (W, C, CC) were housed ad libitum with one of the assigned cage beddings. The mice housed with wooden bedding ate the biggest amount of bedding while mice on cellulose bedding ate the least of bedding (all groups p < 0.001; Fig. 1a). With similar starting bodyweight (Supplementary Fig. S1), overnight-fasted mice from ON-W, ON-C, ON-CC, ON-NB groups lost 13–14% body weight (Fig. 1b) while ON-G mice lost 16% body weight suggesting the impact of coprophagy and/or cage bedding consumption on body weight. CR mice lost 20–22% body weight within 14 days with the CR–CC group losing the most. At the same time, all ad libitum mice gained 6–8% body weight. There were no differences in the weight of the stomach with its content between the ad libitum and CR groups (Fig. 1c) indicating comparable consumption in hours prior to the dissection, even though CR mice had access to food for the last time the evening preceding the dissection. Mice from all ON groups had lighter stomachs than ad libitum fed and CR mice proving diminished consumption (W vs ON-W p = 0.004, C vs ON-C p = 0.006). Both ON-G (ON-W vs ON-G p = 0.002, ON-C vs ON-G p < 0.001, ON-CC vs ON-G p < 0.001) and ON-NB (ON-CC vs ON-NB p = 0.002) groups had smaller stomachs than other ON groups suggesting the lowest food intake. Among ON fasted groups, CC group had the heaviest stomach and its content implying increased consumption or slower digestibility of CC bedding compared to W and C. CR mice, which experienced long-lasting dietary restriction, had heavier stomach with its content than ON mice suggesting that long-term food restriction enhances supplementation with cage bedding and/or feces. Mice from all CR groups showed an increased weight of cecum with its content compared to ad libitum mice (Fig. 1d) pointing toward an accumulation of indigestible fiber. The cecum of the CR-CC group was significantly smaller than the cecum of CR-W mice (p = 0.005) suggesting less fiber deposition. All ON fast mice showed decreased cecum weight compared to ad libitum mice. ON-G group had a statistically significantly lighter cecum than any other ON mice (ON-G vs ON-W p < 0.01, ON-G vs ON-C p < 0.01, ON-G vs ON-CC p < 0.01, ON-G vs ON-NB p = 0.007). All CR and ON mice had smaller liver than corresponding control mice (Supplementary Fig. S1). However, there was no impact of the type of bedding or lack of bedding on the liver size in relation to their body size in ON as well as CR groups. Similarly, CR mice had less epididymal white adipose tissue (eWAT) (Supplementary Fig. S1) and subcutaneous white adipose tissue (sWAT) (Supplementary Fig. S1) than control groups, however, there was no impact of the bedding type. Despite comparable total body weight loss between the different ON groups, the ON-NB group showed higher sWAT content compared to the ON-W, ON-C, and ON-CC groups.

### Cage bedding impacts ghrelin and glucose levels in plasma

Mice from CR and ON groups showed increased plasma total ghrelin levels compared to ad libitum groups (Fig. 1e; all groups p < 0.005). CR-W had a lower level of total ghrelin compared to CR-CC, however, the difference was not statistically significant (p = 0.06). The more pronounced pattern was measured for active ghrelin with values for CR-W stronger contrasting other CR groups and showing significant difference when compared to CR-C (p = 0.004; Fig. 1f). However, each day of CR, mice from all CR groups took a similar time to reach for a daily food portion suggesting equal hunger level (Supplementary Fig. S2). Additionally, the expression of Neuropeptide Y (Npy), leptin receptor (Lepr), and cholecystokinin receptor (Cckr) genes is modified by CR and ON fasting but not affected by the type of bedding (Supplementary Fig. S2). Concerning the ON groups, the type of bedding or lack thereof did not influence plasma active ghrelin levels (Fig. 1f).

CR and ON groups had generally lower plasma glucose levels compared to ad libitum fed groups (Supplementary Fig. S2). Glucose concentration was lower in ON-NB and ON-G than other ON groups; however, due to multiple groups, not statistically significant in all instances (ON-W vs ON-NB p = 0.008, ON-C vs ON-NB p = 0.03, ON-CC vs ON-NB p = 0.05, ON-W vs ON-G p = 0.01, ON-C vs ON-G p = 0.04, ON-CC vs ON-G p = 0.03). Upon glucose load mice from ON-NB and ON-G groups showed decreased blood glucose levels starting from 30 min after glucose administration (Fig. 1g, Supplementary Fig. S2) suggesting increased uptake in peripheral tissues. The area under the curve of plasma glucose levels was smaller for ON-NB and ON-G compared to other ON groups (ON-W vs ON-NB p = 0.004, ON-C vs ON-NB p = 0.008, ON-CC vs ON-NB p = 0.003, ON-W vs ON-G p = 0.009, ON-C vs ON-G p = 0.01, ON-CC vs ON-G p = 0.004; Supplementary Fig. S2).

### Cage bedding affects energy content, microbiota, and metabolites in CR mice cecum

To investigate the impact on the gastrointestinal tract we measured gene expression in the intestinal mucosa. As we previously published^5^, CR tends to increase the expression of metabolic genes (*Pparaα*, *Acot4*, *Scd1*) and decrease the expression of inflammatory genes (*MyD88*, *Rsad2*, *Oasl1a*, *Irf7*) (Supplementary Fig. S3). However, the type of bedding did not affect gene expression.

**Figure 2:**
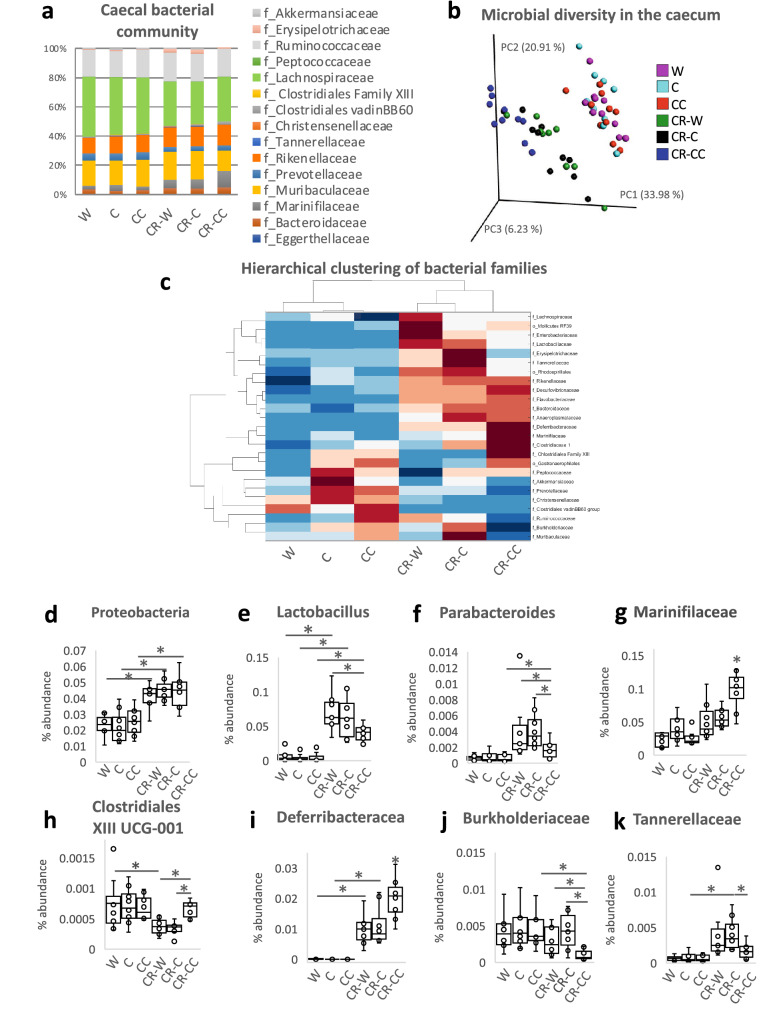
Cage bedding impacts the composition of cecal microbiota. The composition of bacterial families (**a**) and microbial diversity (**b**) in the cecum was analyzed in ad libitum and CR fed mice. The data was presented as a heatmap of the hierarchical clustering analysis of bacterial families using COVAIN (**c**). The abundance of bacteria in the cecum was expressed as % (**d**–**k**). Asterisk (*) indicates statistical significance after Bonferroni correction for multiple testing. Groups were compared using one-way ANOVA. Error bars stand for the mean ± SEM. The data represents nine to ten biological replicates per experimental group.

**Figure 3:**
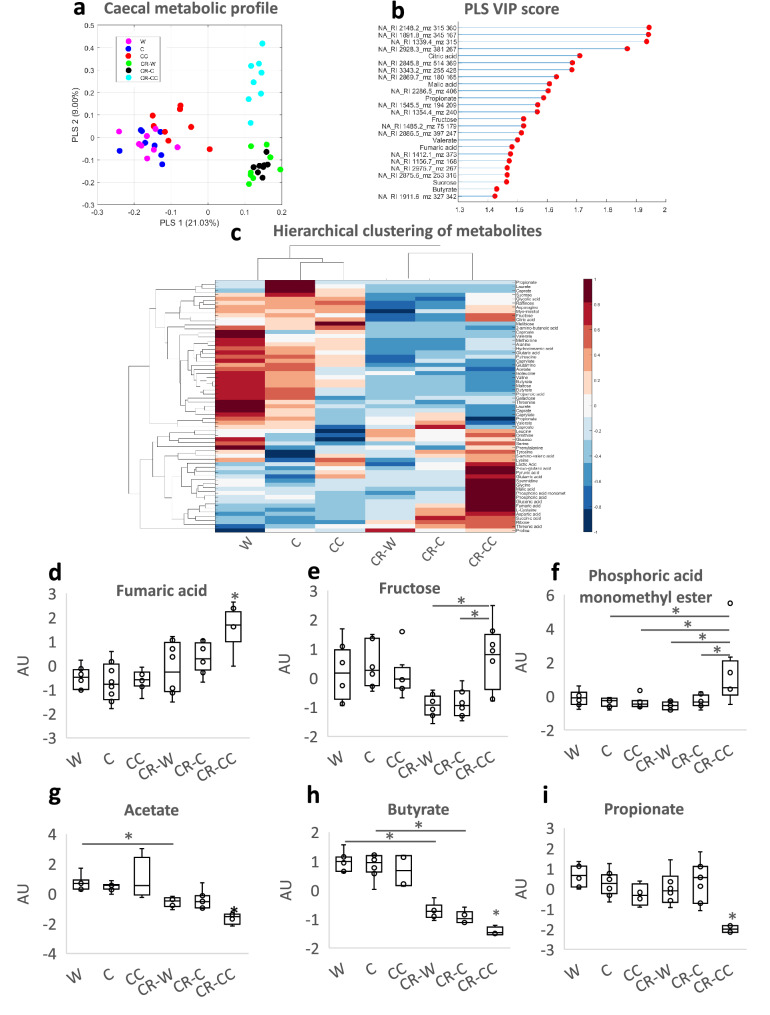
Cage bedding impacts the composition of cecal metabolites. The metabolome of cecal content was analyzed (**a**) and the most important variables were summarized (**b**). Heatmap of hierarchical clustering analysis of annotated metabolites was created using COVAIN (**c**). Z-Scored metabolites figures show the relative deviation from the groups mean value (0) for fumaric acid (**d**), fructose (**e**), and phosphoric acid monomethyl ester (**f**) represent the most important annotated metabolites contributing to a distinct metabolic profile within the CR group. The cecum content of SCFAs was analyzed (**g**–**i**). Single data points are indicated by circles and medians as horizontal lines within each box. One-way ANOVA was applied to verify statistical significance. Asterisk (*) indicates statistical significance after Bonferroni post-hoc analysis. Error bars stand for ± SEM; n = 8–10.

**Figure 4:**
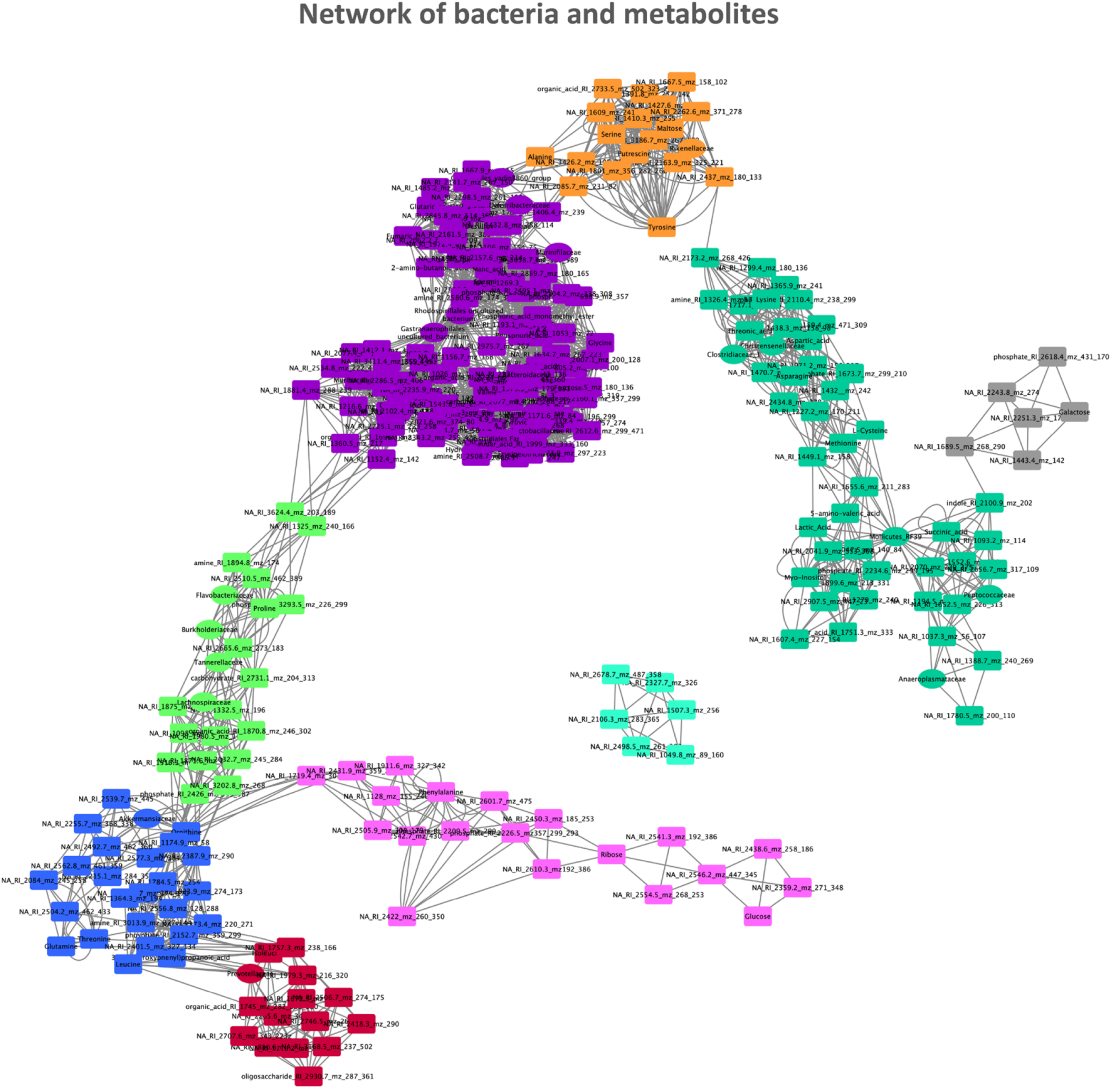
Correlation network of bacteria with metabolites in the cecum. The correlation network depicts changes in bacterial families composition and metabolites occurrence characteristic to CR. Each node represents one metabolite (ellipse) or a bacterial family-level OUT (V-shape) and each edge represents a statistically significant correlation where the Pearson’s correlation coefficient ≥ 0.8. Girven–Newman algorithm was applied in network clustering analysis where modules (clusters, denoted by different colors) depict association patterns between metabolites and bacteria. The visualization was performed with Cytoscape v3.7.2. (http://www.cytoscape.org/).

The bedding was collected from CR mice cages every 24 h for three consecutive days and feces were separated, dried, and weighed. The CR mice produced fewer feces than ad libitum groups (for all groups p < 0.001; Fig. 1h) and the feces contained less energy; however the difference was not statistically significant when correcting for the number of experimental groups (W vs CR-W p = 0.04; Fig. 1i). Importantly, CR-CC produced more feces compared to the other CR groups (for both groups comparison p < 0.001; Fig. 1h). Further, we analyzed cecal microbiota as fermentation of indigestible food in mice is compartmentalized in the cecum. At the same time, microbiota and metabolites share similarities between cecum, colon, and feces^20,21^. All ad libitum fed mice groups shared similar microbial composition (Fig. 2a). We observed a strong shift in cecal bacteria composition from ad libitum to CR mice (Fig. 2a). In general, among the CR groups, CR-W and CR-C bacteria composition overlapped while CR-C was distinct (Fig. 2b-c). CR was accompanied by a non-statistically significant shift in the ratio of *Firmicutes* to *Bacteroidetes* (CC vs CR-CC p = 0.01; Supplementary Fig. S4). The strongest differences on the phylum level were recorded for *Proteobacteria* (all ad libitum vs CR groups p < 0.001; Fig. 2d, Supplementary Fig. S4) and *Deferribacteres* (all ad libitum vs CR groups p < 0.001; Supplementary Fig. S4). The sequencing results were compared to published CR reports^22–26^ and the previously observed decrease in abundance of *Roseburia* (C vs CR-C p = 0.004, CC vs CR-CC p < 0.055), *Butyricicoccus* (all ad libitum vs CR groups p < 0.001), *Streptococcus* (W vs CR-W p = 0.005, C vs CR-C p < 0.001, CC vs CR-CC p < 0.001)*, Anaerotruncus* (C vs CR-C p = 0.001), and *Lachnospiraceae* (all ad libitum vs CR groups p < 0.001; Supplementary Fig. S4) as well as an increase in *Lactobacillus* (all ad libitum vs CR groups p < 0.001), *Parabacteroides* (W vs CR-W p = 0.02, C vs CR-C p < 0.001, CC vs CR-CC p < 0.01)*,* and *Odoribacter* (W vs CR-W p = 0.02, C vs CR-C p = 0.03, CC vs CR-CC p < 0.001) in CR compared to ad libitum (Fig. 2e,f, Supplementary Fig. S4) were observed. In other reported CR-affected Operational Taxonomic Units (OTU) (e.g. *Alistipes*, *Alloprevotella*, *Erysipelotrichaceae*, *Intestinimonas*, *Lachnoclostridium*, *Marvinbryantia*, *Roseburia*, *Ruminococcaceae*)^23–26^ we observed inverse or no effect of CR (Supplementary Fig. S5). Importantly, there has also been little overlap between different published data sets. We identified phylum *Deferribacteres* (CR-W vs CR-CC p < 0.001, CR-C vs CR-CC p = 0.003), genus *Lactobacillus* (CR-W vs CR-CC p < 0.006, CR-C vs CR-CC p = 0.03) as well as the families *Marinifilaceae* (CR-W vs CR-CC p < 0.001, CR-C vs CR-CC p < 0.001), *Clostridiales XIII* UCG-001 (CR-W vs CR-CC p < 0.001, CR-C vs CR-CC p < 0.001), *Deferribacteraceae* (CR-W vs CR-CC p < 0.001, CR-C vs CR-CC p = 0.008)*, Burkholderiaceae* (CR-W vs CR-CC p = 0.009, CR-C vs CR-CC p < 0.001), and *Tannerellaceae* (CR-C vs CR-CC p = 0.008) as bacteria affected the strongest (based on *p*-value) by the bedding type in CR mice, particularly by the difference between CC versus C and W beddings (Fig. 2e,g–k, Supplementary Fig. S4). Moreover, OTUs *Odoribacter* (CR-W vs CR-CC p < 0.001, CR-C vs CR-CC p < 0.001), *Mucispirillum* (phylum *Deferribacteres)* (CR-W vs CR-CC p < 0.001, CR-C vs CR-CC p = 0.003), *Parasutterella* (CR-W vs CR-CC p = 0.02, CR-C vs CR-CC p = 0.002), and *Erysipelatoclostridium* (CR-W vs CR-CC p < 0.001, CR-C vs CR-CC p = 0.007), *Eubacterium* (from *Xylanophilum* group; CR-W vs CR-CC p = 0.019, CR-C vs CR-CC p = 0.007), *Ruminoclostridium 6* (CR-W vs CR-CC p = 0.03, CR-C vs CR-CC p = 0.002) and *Ruminococcus 1* (CR-W vs CR-CC p = 0.05, CR-C vs CR-CC p = 0.001) distinguished CR-CC from CR-W and CR-C (Supplementary Figs. S4 and S6). Correspondingly, the composition of cecal metabolites was affected by the restriction and bedding type (Fig. 3a). The metabolites composition was similar in the mice groups fed ad libitum while among CR groups, CR-W and CR-C were distinct from CR-CC. Variable importance for prediction (VIP) scores were calculated from the PLS and the top 25 highly significant metabolites that cause the difference between the groups have been identified (Fig. 3b). The hierarchical clustering of metabolites further visualized the differences between the groups (Fig. 3c). Among the metabolites setting the CR groups apart, fumaric acid (CR-W vs CR-CC p = 0.03, CR-C vs CR-CC p = 0.002), fructose (CR-W vs CR-CC p = 0.001, CR-C vs CR-CC p = 0.001), and phosphoric acid monomethyl ester (CR-W vs CR-CC p = 0.02, CR-C vs CR-CC p = 0.001) were identified (Fig. 3c-f). The levels of SCFAs acetate (CR-W vs CR-CC p = 0.01, CR-C vs CR-CC p = 0.006) and butyrate (CR-W vs CR-CC p = 0.001, CR-C vs CR-CC p = 0.002), as well as medium-chain fatty acids (MCFAs), were decreased in all CR groups regardless of the type of bedding (Fig. 3g–i, Supplementary Fig. S7). The levels of propionate were significantly downregulated only for the CR-CC group (CC vs CR-CC p = 0.001, CR-W vs CR-CC p = 0.001, CR-C vs CR-CC p = 0.001; Fig. 3i). Further, we sought to identify which bacteria could contribute to the observed metabolomic changes. A correlation between changes in bacteria composition and metabolites occurrence revealed the co-dependence of multiple factors (Fig. 4, Table 1). Correlation coefficients were calculated for the CR groups and depicted by Cytoscape into clusterings of cecal bacterial families and metabolites indicating strong interaction (Fig. 4).

**Table 1:**
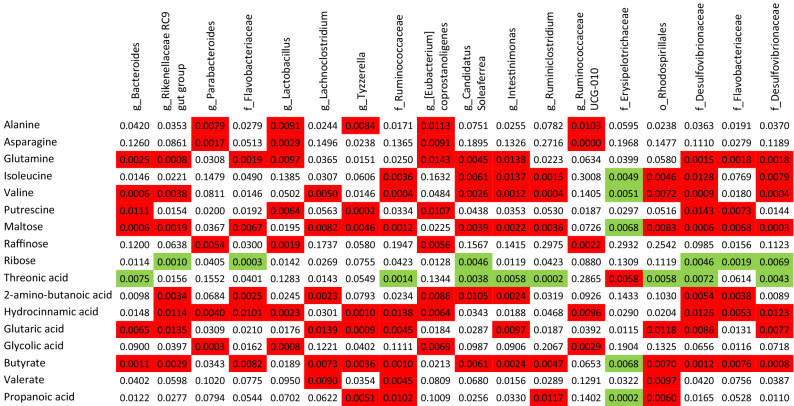
Correlation of bacteria with metabolites in the cecum.

Correlation p-values between annotated metabolites and the bacterial genus in the cecum of CR mice were calculated. For Operational Taxonomic Units (OTU) that could not be assigned on the genus level, the closest taxonomical level of identification was used. Characters before the name of the bacteria represent family (f), genus (g), and order (o). Coloured cells show statistically significant differences; red = negative correlation; green = positive correlation. Correlation coefficient analysis using Pearson’s correlation (r = 0.8) was done in COVAIN; p < 0.0083.

## Discussion

Previously we showed that mice submitted to CR increased their cecum weight as a likely result of the accumulation of indigestible fiber from bedding^5^. In the current project, we investigated the consequences of the increased consumption of cage bedding and how it influences the outcome of animal studies. We show the impact of cage bedding in restrictive dietary protocols, reflected by differences in body weight, adiposity, cecum size, glucose, and ghrelin levels in plasma as well as cecal microbiota and metabolites profile.

All CR mice consumed bedding, however, the amounts of eaten beddings varied suggesting a difference in preference (olfactory and gustatory properties), the capacity of the bedding to deliver energy, or offer satiety. The CR groups showed bedding-dependent variability in weight loss. The difference may be explained by the availability of fiber which, especially the soluble one, can serve as a source of energy (2 kcal/100 g)^27^. CR-W and CR-C groups consumed mostly cellulose which is fermentable to some extent (up to 70%—80%), even in humans^28,29^. Similarly, hemicellulose can be bacterially digested^29^ and it might improve the fermentability of cellulose^30^. While lignin is indigestible by human enzymes^29^, it was shown in vitro to be partially digestible by the human microbiome^31^ and in vivo by rats^32^. In order to compare the amount of energy taken up and extracted by mice from the ingested beddings energy content in feces was measured. We were not able to detect differences in fecal energy content (kJ/g) between the CR groups, however, it is important to notice that the CR-CC group produced more feces (g/day) and therefore secreted more total energy (kJ/day). This result is accompanied by the highest body weight loss for the CR-CC group. Therefore, we suspect that of the three beddings CC was the source of which the mice were able to absorb the least of energy. Importantly, it is impossible to state whether the differences in the measured amounts of feces result from disparities in the production or the consumption of feces.

The weight of the stomach with its content was considered in our study as an indicator of the scale of consumption within the last hours prior to the dissection. The smaller stomach size in the animals housed on the grids proved our concept. Interestingly, although ON fasted animals consumed bedding their stomachs were much smaller than the stomachs of CR mice, whereas the difference between ad libitum and CR mice was minor. Therefore, prolonged restriction favors enhanced bedding consumption. Similarly to the stomach, the weight of the cecum with its content reflected the amount of fiber consumption. However, this was more specific in a way that it also pictured the intake over a prolonged period, fiber accumulation, and digestibility of the fiber in the given beddings. Interestingly, the size of the cecum reversely correlated with the amount of feces produced as CR mice had a smaller cecum when housed on CC bedding.

Since bedding and feces ingestion influence the outcomes of metabolic studies^14,33,34^ rodents may be kept on a metal grid short-term^35^ to prevent bedding consumption as well as limit coprophagy. However, this experimental setup cannot stop mice from eating feces directly from the anus. To completely cease coprophagy, the use of anal cups or neck restrainers would be necessary, which would be distressing to the animal and could interfere with normal feeding behavior. In our study, mice housed on the grid showed higher body weight loss as well as lighter stomach and cecum compared to conventionally fasted mice, indicating less consumption. It has previously been shown that rats housed in wire-bottom cages do not show any clinical pathology symptoms when compared to rats from solid bottom cages^36^. However, housing rats in wire-bottom cages overnight leads to immediate alterations of heart rate, body weight, and locomotor activity, which might be related to stress response^37^. Therefore, the bodyweight loss in the mice fasted on the grid may also indicate the impact of additional stress or energy loss required for body temperature regulation when deprived of nesting material. When coprophagy was permitted, ON fasted mice did not differ in cecum and body weight from mice with access to bedding indicating the importance and scale of coprophagy. Coprophagy occurs not only to compensate for energy during deprivation but also to supplement the diet with various nutrients of gut microbiota origin. Choline, cysteine, thiamine, iron, vitamin K, B_12_, and essential fatty acids are sourced from feces and if the animals are not allowed to consume feces, chow needs to be supplemented^38–46^. However, on an ad libitum balanced laboratory diet coprophagy might not be crucial, as its absence does not result in lower body weight or less progeny^47^. Most importantly, coprophagy leads to the re-inoculation of the gut, therefore, it is an important factor in microbiota composition. Moreover, bedding presence and not coprophagy was the deciding factor concerning glucose tolerance. Both groups, housed without bedding or kept on the grid showed lower glucose levels compared to any group submitted to ON in the presence of bedding despite the fact that the initial, fasting glucose levels were comparable between all the groups.

Since CR results in hunger, the impact of supplementation with different beddings on hunger was tested by assessing the speed of meal initiation. Bedding did not influence how rapidly the mice started consumption of the daily portion of chow. Nevertheless, after day two of CR, all groups ate nearly immediately following access to food, therefore, beyond this point small differences in the speed of the meal initiation were difficult to detect. Moreover, basal blood glucose levels and gene expression in the hypothalamus were comparable between the animals of different CR and ON groups. Importantly, the levels of active ghrelin, the “hunger hormone”, were increased in CR-CC and CR-C compared to CR-W. This indicates that the type of bedding may influence hunger perception.

Several publications reported changes in microbiota composition in CR compared to ad libitum fed mice. Data comparison revealed a few similarities of our results with previous studies^22–26^ concerning consistent trends in the abundance of selected OTUs, regardless of the type of bedding. Nevertheless, numerous differences in bacteria occurrence were found between ours and the published sequencing results. Obviously, microbiota composition depends on multiple factors including different types of cage bedding, contributing to the differences in outcomes of similar experiments. Accordingly, strong differences in gut bacteria and metabolites were observed between beddings having the most distinct fiber profile (CC vs C and W), while C and W bedding, which stem from the same initial material, resulted in comparable microbiota composition. Fittingly, *Ruminococcus,* which abundance raises in response to high-cellulose diet^48^, was increased in CR-C and CR-W versus corresponding ad libitum groups but this was not present in CR-CC. Consequently, *Ruminococcus* was identified as one of the OTUs distinguishing CR-CC versus both CR-W and CR-C. Therefore, it is important to consider that the main trigger of microbiota composition changes in CR animal trials is not the nutrient restriction but the bedding consumption.

*Lactobacillus* which is known to mitigate inflammation and improve gut barrier function is stimulated by CR^22,49^. Also in our study CR strongly increased the abundance of *Lactobacillus* in mice cecum. This result corresponds well with our previous report^5^ showing a decrease in expression of the immune function-related gene in the intestine of CR mice. Importantly, the type of bedding affected the extent to which the bacteria levels increased, therefore, likely modulating inflammatory status.

A high-fiber diet affects body weight, GI-tract and liver status, microbiota composition, fiber fermentation, and general health^8,50,51^. However, most of the studies point toward a stronger impact of soluble versus insoluble fiber since these are the vital substrates for SCFA production^16,50,52,53^. Despite high fiber intake and cecum enlargement suggesting an elevated fermentation, CR mice showed lowered levels of all short to medium-chain fatty acids. The lowered levels of SCFA may be associated with the decrease in the relative abundance of enzymes involved in butyrogenesis and acetogenesis upon CR^23^. However, an increase in propiogenesis-related enzymes has been reported in parallel^23^. Moreover, supplementation with fiber including cellulose or corn fiber does not result in an increased SCFA in the cecum^54,55^. The knowledge concerning insoluble fiber fermentation by gut microbiota is very limited. In general, compared to soluble fiber, cellulose is poorly fermented, it yields mostly acetate and less propionate or butyrate^54,55^, and the process takes place mostly in the distal colon where transit time is slower, and bacterial densities are higher^56^. There are multiple fecal strains digesting cellulose from both major phyla *Bacterioidetes* and *Firmicutes*^57^ and the main cellulolytic strains isolated from human feces have been classified as *Ruminococcus* sp, *Clostridium* sp, *Eubacterium* sp, and *Bacteroides* sp.^57–60^. Therefore, despite the lower fermentability of cellulose compared to soluble fiber, we assume that SCFA production increased in CR mice supplementing themselves with cage bedding. We propose that the produced metabolites are likely taken up more efficiently and utilized by the host due to CR-related energy shortage. Additionally, the levels of fumaric acid, an intermediate product of bacterial fermentation are increased in all CR groups and highest in CR-CC. This implies an increased fermentation particularly strongly stimulated by CC bedding.

We analyzed the correlation between the occurrence of the detected cecal microbiota and corresponding metabolites. We could confirm the previously published negative correlation between *Bacteroides* and fatty acids^61,62^*, Oscillibacter* and isoleucine^63^ and positive correlation between *Alistipes* and proline^62^, *Lachnoclostridum* and glutaric acid^64^, *Bacteroides* and spermidine^65^ as well as *Blautia* and malic acid and myo-inositol^62^. However, most of the observed correlations have not been reported before.

To summarize, based on the differences between mice kept with or without cage bedding we can conclude that mice consume bedding and feces during dietary restrictions and this influences their body, WAT, stomach and cecum weight, cecal microbiota and metabolites profile as well as plasma glucose and ghrelin level. Therefore, we propose that the amount of energy extracted from fiber depends on the bedding type and contributes to the bodyweight differences. Moreover, in the to-date published reports on the effect of CR, particularly on microbiota, in mice or rats, it is impossible to distinguish between the effect of CR or the supplemented bedding making the results not comparable to human CR studies. The reproducibility of published results is a major issue in the scientific community. Our data indicate an important factor that needs to be taken into account when interpreting and designing experiments, particularly important when restrictive diets are considered. With great progress in the field of microbiota within the last few years and knowing the importance of dietary fiber as a source of prebiotics, it is important to indicate that gut bacteria composition is affected by the type of fiber present in mice cage bedding. We propose that for short-term restrictions, cages with grid floors are used. Due to ethical concerns, this approach cannot be applied to long-term experiments, instead it should be encouraged to routinely report not only the type of the diet but also cage bedding.

## Materials and methods

### Animal experiments

Male C57BL/6NRj mice purchased from Janvier Inc. Labs (Le Genest, France) were housed in standard SPF conditions using a Tecniplast IVC system (cage type 2L, blue line). Mice were fed a standard chow (V1535 R/M-H Extrudate; ssniff Spezialdiäten GmbH, Soest, Germany). The animals were divided into control ad libitum, CR, and ON fast groups (Supplementary Table S1). Each of these groups was separated into three subgroups by the bedding type: wooden (Lignocel select), corncob (RehoFix MK 3500), cellulose (Arborcel Performance Small; all beddings from J. Rettenmaier & Söhne GmbH + Co KG; Vienna, Austria). The fiber composition of the beddings is presented in the Supplementary Fig. S1A. Additionally, two subgroups of the ON fast group were housed without any cage bedding or on a metal grid to prevent contact with cage bottom and coprophagy. The control group mice were kept ad libitum on each of the bedding. Each group of mice contained 10 animals; however, during the dissection some of the tissues were lost resulting in 8–10 replicates for the presented results. Mice from CR groups were submitted to two weeks CR with 75% of normal food intake. Mice body weight was measured daily during the CR protocol. To estimate the hunger of the CR mice, a daily food pellet was placed in the cage and the time it took the mice to initiate the meal was measured with a stopwatch. Cage bedding was changed daily from the 11^th^ day of CR for three consecutive days to assess the amount of bedding eaten. The harvested bedding was dried and feces were separated. The feces were dried and used for verification of daily fecal mass production and energy content measurement by direct calorimetry (IKA-Kalorimeter C2000; IKA-Werke GmbH & Co. KG; Staufen, Germany). Fresh fecal samples were collected on days 12 and 13 of CR and from ad libitum fed mice. The feces were snap-frozen and stored at − 80 °C.

For the ON fasted groups, food was removed in the evening and the mice were fasted 16 h with free water access. After ON fast, mice were submitted to an oral glucose tolerance test (OGTT) by gavaging 3 mg glucose per gram body weight. Afterward, the mice were fed ad libitum. One week later the ON fasting procedure was repeated and mice were sacrificed and dissected in the morning. Food from the cages of control mice was removed 2 h before the dissection. All mice were euthanized by isoflurane overdose, with blood drawn by cardiac puncture. The stomach and cecum with their content as well as adipose tissue and liver weight were recorded. Blood was mixed with 10 μl/ml EDTA, 20 μl/ml aprotinin, and 10 μl/ml dipeptidyl peptidase (DPP) IV. Plasma was separated from the blood cells by centrifugation for 10 min at 3,600xg 4 °C and was stored at − 80 °C. Stomach, small intestine, and colon scrapings as well as cecum content were snap-frozen and stored at − 80 °C until use.

All animal experimentation protocols were approved by the Federal Ministry of Science, Research and Economy, Unit for Animal Experiments and Genetic Engineering in Austria (BMWFW-66.006/0017-WF/V/3b/2016). The experiments were performed in agreement with the Austrian Federal Act on Animal Welfare.

### Sequencing the 16S rDNA genes and metataxonomic analysis

The samples for sequencing were processed according to the previously published protocol^66^. Cecum samples were homogenized in MagNA Pure Bacteria Lysis Buffer from the MagNA Pure LC DNA Isolation Kit III (Bacteria, Fungi) in MagNA Lyser green beads tubes at 6,500 rpm for three 30 s cycles in a MagNA Lyser Instrument (all from Roche, Mannheim, Germany). The homogenized samples were mixed with 25 μl lysozyme (100 mg/ml), incubated at 37 °C for 30 min followed by adding 43.4 μl Proteinase K (20 mg/ml) and incubation at 65 °C overnight. Afterwards, the enzymes were heat-inactivated at 95 °C for 10 min and 250 μl lysed supernatant was used for DNA extracted on a MagNA Pure LC 2.0 following the instructions for the MagNA Pure LC DNA Isolation Kit III (Bacteria, Fungi) (Roche). PCRs reactions were run in triplicates using a FastStart High Fidelity PCR system and contained 5 μl of total DNA, 1 × Fast Start High Fidelity Buffer, 1.25 U High Fidelity Enzyme, 200 μM dNTPs, 0.4 μM primers, and PCR-grade water in 25 μl reaction volume (all reagents from Roche, Mannheim, Germany). The following target primers were applied for the amplification of phylogenetic informative hypervariable regions V1-V2: 27F—AGAGTTTGATCCTGGCTCAG and 375R—CTGCTGCCTYCCGTA. The primers were used with Illumina adapters for indexing PCR reaction according to Illumina's 16S metagenomic sequencing library preparation guide. The PCR temperature cycles were as follows: initial denaturation at 95 °C for 3 min, 30 cycles of denaturation at 95 °C for 45 s, annealing of primers at 55 °C for 45 s and extension at 72 °C for 1 min, final extension step at 72 °C for 7 min and cooling to 4 °C. The PCR reaction triplicates were pooled and checked using 1% agarose gel and subsequent normalization of 20 μl PCR products was performed on a SequalPrep Normalization Plate (LifeTechnologies, Germany). Of the normalized PCR products, 15 μl was used as a template in a single 50 μl indexing PCR reaction for 8 cycles; the temperature cycles conditions were as described above for the targeted PCR. For the final sequencing library, 5 μl of PCR products from each sample were pooled and 30 μl of the library was purified using a 1% agarose gel and the QIAquick gel extraction kit (Qiagen, Germany). The obtained library was quantified with QuantiFluor ONE dsDNA Dye on Quantus Fluorometer (Promega, Germany), its quality was verified using an Agilent BioAnalyzer 2100 (Waldbronn, Germany) and the 6 pM library was sequenced on a MiSeq desktop sequencer (Illumina, Netherlands) containing 20% PhiX control DNA (Illumina) with v2 chemistry for 500 cycles. FastQ raw reads were used for subsequent data analysis.

Raw sequencing data in fastq format was imported in Galaxy web-based platform^67^ and analyzed with the QIIME2 2018.4 microbiome analysis pipeline. After initial quality control data was preprocessed with DADA2^68^ using default parameters and removing specific primer sequences. The resulting feature representative sequences were classified with the QIIME2 pre-fitted sklearn-based taxonomy classifier against SILVA 16S rRNA database version 132 at 99% identity^69^. The resulting feature abundance table, also known as OTUs table over all samples including taxonomy information was used for all subsequent analyses. For the phylogenetic methods, representative sequences were aligned with MAFFT de novo multiple sequence aligner^70^ followed by the creation of a phylogenetic tree with FastTree^71^.

### Metabolomics

Extraction and analysis of cecal metabolites were performed according to Weckwerth et al.^72^ with slight modifications. Frozen samples (~ 30 mg) were transferred into “Precellys lysis kit” homogenizing tubes with 1.4 mm ceramic beads and 800 μl ice-cold MCW extraction buffer (methanol:chloroform:water = 2.5:1:0.5) was added. The samples were homogenized in a Precellys24 Tissue Homogenizer (Bertin Instruments) twice for 15 s at 5000 rpm and were incubated on ice for 15 min. Next, samples were vortexed and centrifuged for 5 min at 10,500 rpm at 4 °C then the supernatant was transferred to a 2 ml Eppendorf tube. The extraction step was repeated by short vortexing the pellet with 400 μl ice-cold MCW followed with 15 min incubation on ice and centrifugation for 5 min at 10,500 rpm at 4 °C. The two supernatants were combined and to separate chloroform phase from the water/methanol phase 400 μl H_2_O was added. After vortexing and centrifuging the samples for another 5 min at 14,000 rpm at 4 °C, the upper polar phase was transferred to a new Eppendorf tube and both fractions were dried in a speed vac using an optimized pressure gradient to prevent boiling retardation. The polar fraction was dissolved in 50 μl of methoxamine hydrochloride solution (20 mg/ml pyridine) and incubated at 30 °C for 90 min with continuous shaking. Then 80 ml of *N*-methyl-*N*-trimethylsilyltrifluoroacetamid (MSTFA) was added to derivatize polar functional groups at 37 °C for 30 min. The derivatized samples were stored at room temperature for 120 min before injection. Gas chromatography–mass spectrometry (GC–MS) analysis was performed using a Leco Pegasus BT-TOF (Leco Instrumente GmbH, Mönchengladbach, Germany) equipped with a PAL3 Autosampler (CTC Analytics AG, Zwingen, Switzerland). Chromatographic separation and data validation were conducted as published earlier with slight modifications^73,74^. Derivatized extract (1 µl) was injected on an HP-5MS column (30 m × 0.25 mm × 0.25 μm) (Agilent Technologies) in split less mode. Mass spectral data acquisition was performed using the following instrument parameters. Electron impact ionization was conducted at 70 eV and 1 mA emission current. Ion source and transferline temperature were set to 250 °C. Mass spectra were collected at an acquisition rate of 10 spectra/sec and a mass range of 40–600 Th using a relative detector voltage with an offset of − 100 V from optimized detector voltage. Mass spectrometry data are stored at MetaboLights (https://www.ebi.ac.uk/metabolights/).

### Statistical analysis

OTUs table was reduced by removing all OTUs present in less than three samples per group. The obtained data of GC–MS and LC–MS were normalized to fresh weight, then annotated and classified according to the Metabolomics Standards Initiative (MSI). Data transformation, alignment, and integrative analysis including correlation coefficient, partial least square (PLS) regression, one-way ANOVA, hierarchical clustering, and correlation network analysis were performed with the statistical software COVAIN^75^ under MATLAB environment. The amount of each metabolite and bacteria OTUs were z-scored across all samples. The correlation network associating metabolites (classified as MSI 1 and 2) and bacteria OTUs was constructed by Pearson’s correlation coefficients (cutoff value = 0.8). For network visualization, Girven-Newman algorithm^76^ was applied and visualization was performed with Cytoscape v3.7.2. (http://www.cytoscape.org/).

Concerning other data sets, the experimental groups were compared applying one-way ANOVA with Bonferroni post-hoc corrections for multiple testing. Where applicable, differences between two experimental groups were analyzed using Student’s t-test with statistical significance threshold set at p < 0.05. Each of the groups contained 8–10 biological replicates.

## Supplementary information

Supplementary Information 1.

Supplementary Information 2.

## Data Availability

The microbiota and metabolomics datasets generated during and analyzed during the current study are available in the European Nucleotide Archive [https://www.ebi.ac.uk/ena/browser/view/PRJEB37837] and MetaboLights repository, [www.ebi.ac.uk/metabolights/MTBLS1631] respectively.
