# Supplementary material for: Cage bedding modifies metabolic and gut microbiota profiles in mouse studies applying dietary restriction
Source: Sci Rep. 2020 Nov 30;10:20835. doi: 10.1038/s41598-020-77831-3 (PMC7705694; doi:10.1038/s41598-020-77831-3)
Supplement: Supplementary file 1 — Supplementary Information 1. [file 41598_2020_77831_MOESM1_ESM.pdf]

Cage bedding modifies the outcomes of metabolic and microbiota studies

Gregor A, Fragner L, Trajanoski S, Li Weimin, Sun Xiaoliang, Weckwerth W, König J, Duszka K

Supplementary information

Supplementary table S1. Experimental groups.

Control/restriction	Bedding type	Group name
control	Wooden	W
control	Cellulose	C
control	Corncob	CC
CR	Wooden	CR-W
CR	Cellulose	CR-C
CR	Corncob	CR-CC
ON fast	Wooden	ON-W
ON fast	Cellulose	ON-C
ON fast	Corncob	ON-CC
ON fast	No bedding	ON-NB
ON fast	No bedding, metal grid	ON-G

Supplementary materials and methods

qRT-PCR

RNA was isolated from intestinal scrapings using the RNeasy mini kit (Qiagen, Hilden, Germany). Samples were thawed in lysis buffer, disrupted using a syringe and needle, and processed following the manufacturer's recommendations. qScript cDNA synthesis kit SuperScript® II Reverse Transcriptase (Quanta Biosciences, MA, USA) was used for the reverse transcription step. Quantitative real-time PCR (qRT-PCR) reactions were carried out using the QuantaStudio 6 Flex (Applied Biosciences, CA, USA) with the PerfeCTa® SYBR® Green PCR Master Mix (Quanta Biosciences). Primers for NPY and LEPRP were purchased form Qiagen. Other primers used are listed below.

	Forward	Reverse
ACOT4	TGCGGTACATGCTTCGACAT	TGGAAACTGTGGCTGAGACAT
CCKR	AGTGAGCCATTCACCAGCTC	CACTGCAGACTGCCATTCTTT
IRF7	GAGACTGGCTATTGGGGGAG	GACCGAAATGCTTCCAGGG
MyD88	GCACCTGTGTCTGGTCCATT	TGTTGGACACCTGGAGACAG
OAS1A	ATGGAGCACGGACTCAGGA	TCACACACGACATTGACGGC
PPARα	TACTGCCGTTTTCACAAGTGC	AGGTCGTGTTCACAGGTAAGA
RSAD2	TGCTGGCTGAGAATAGCATTAGG	GCTGAGTGCTGTTCCCATCT
SCD1	GCCCACATGCTCCAAGAGAT	GGGCACTGTCTTCACCTTCT
STAT1	CAGTATGATGAGCACAGTA	AAGTCCTTCAGAGTAACAG

Supplementary table S2. qRT-PCR primers.

Plasma ghrelin analysis

Plasma total and active ghrelin were measured in plasma using ELISA (Millipore) following the manufacturer's indication.

Oral glucose tolerance test (OGTT)

OGTT was performed following ON fasting in cages with corresponding bedding, without bedding or on metal grids. Mice tail tip was cut and base glucose levels were measured at -15 min, 0 min, 15 min, 30 min, 45 min, 60 min, 90 min, 120 min, and 150 min time points. The mice were delivered intragastric bolus of glucose 3mg per gram body weight. Blood glucose levels were measured at the indicated time points.

SCFA and MCFA analysis

The detection method was developed based on publications using LC-MS for the analysis of 2-NPH or 3-NPH derivatized fatty acids [1-3]. Frozen tissue samples (~30 mg) were transferred into Precellys homogenizing tubes with 1.4 mm ceramic beads and 800 µl ice-cold extraction buffer (methanol : chloroform : water = 2.5 : 1 : 0.5) was added. The samples were homogenized in the "Precellys 24 homogenizer" twice for 15 seconds at 5,000 rpm, vortexed for 1 min, and were incubated on ice for 15 minutes on a laboratory rocker. Next, the samples were vortexed and centrifuged for 5 min at 10,000 rpm at 4°C. The supernatant (600 µl) was transferred to a 2 ml Eppendorf tube. The extraction step was repeated by vortexing the pellet for 1min with 400 µl ice-cold extraction buffer followed with 15 min incubation on ice on a laboratory rocker and centrifugation for 5 min at 10,000 rpm at 4°C. The supernatants from the two extractions were combined, shortly vortexed and centrifuged for 5 min at 14,000 rpm at 4°C. 100 µl of each sample was transferred to a new 1.5 ml Eppendorf tube, was dried in SpeedVac concentrator for 60 min at 45°C. If not derivatized instantly, samples were stored at -80°C until further processing.

For derivatization, samples were resuspended in 150 µl acetonitrile : water (1:1) and 40 µl 40 mM 2-NPH, 40 µl 250 mM EDC and 40 µl 3% Pyridine was added followed by vortexing and incubation for 30 min at 60°C. Samples were left at room temperature to cool down, afterward put on ice for 2 min and then vortexed. After repeating the 2 min incubation on ice and vortexing, samples were centrifuged at 14000 rpm at 4°C and were dried in the SpeedVac concentrator for 75 min at 45°C. Samples were stored over-night at -20°C and, to avoid phase separation, were resuspended first in 50 µl acetonitrile : water (1:1) then another 50 µl of acetonitrile : water (9:1) was added. Samples were vortexed thoroughly and were transferred into HPLC vials. Samples (10 µl) were directly injected from a thermostatic autosampler kept

at 10°C and analyzed by liquid chromatography coupled to mass spectrometry (LC-MS) using an Ultimate 3000 (Thermo Fischer Scientific, Waltham, Massachusetts, US) and a micrOTOF-Q II (Bruker Daltonics, Bremen, Germany) with an Atlantis T3 3 µm column (2.1x150mm, Waters, Milford, MA, USA). The column temperature was 40°C. The ratio of mobile phase B (acetonitrile + 0.1% formic acid) was slowly increased from 5% (0-2.5 min) to 90% (8 min), followed with a 5 min hold at 90%, then the column was washed with 95% mobile phase A (H_2O + 0.1% formic acid) for 2 min. The retention time of acetate, propionate, butyrate, valerate, caproate, caprylate, caprate, and laurate was 8.0 min, 8.6 min, 9.0 min, 9.5 min, 10.0 min, 10.9 min, 12.3 min, and 14.9 min, respectively. In ESI negative mode the precursor ion of acetate, propionate, butyrate, valerate, caproate, caprylate, caprate, and laurate was was 194.05m/z, 208.07 m/z, 222.08 m/z, 236.10 m/z, 250.12 m/z, 278.15 m/z, 306.18 m/z and 334.21 m/z, respectively.

Chemicals

Solvents and chemicals were purchased from VWR Chemical (Fontenay-sous-Bois, France) or Sigma-Aldrich (Steinheim, Germany). 1,2,3,4-13C-labelled caprylate was from Cambridge Isotope Laboratories (Tewksbury, USA) and 4-APEBA from AxonMedChem (Groningen, Netherlands). The mobile phases A and B used in this work were made of a mixture of water (A=95%, B=5%), methanol (A=5%, B=95%) and formic acid (0.1%). If not stated otherwise, all the solutions were prepared with ddH_2O.

1 Peters, R., Hellenbrand, J., Mengerink, Y. & Van der Wal, S. On-line determination of carboxylic acids, aldehydes and ketones by high-performance liquid chromatography-diode array detection-atmospheric pressure chemical ionisation mass spectrometry after derivatization with 2-nitrophenylhydrazine. J Chromatogr A 1031, 35-50 (2004).

2 Han, J., Lin, K., Sequeira, C. & Borchers, C. H. An isotope-labeled chemical derivatization method for the quantitation of short-chain fatty acids in human feces by liquid chromatography-tandem mass spectrometry. Anal Chim Acta 854, 86-94 (2015).

3 Torii, T. *et al.* Measurement of short-chain fatty acids in human faeces using high-performance liquid chromatography: specimen stability. Ann Clin Biochem 47, 447-452 (2010).

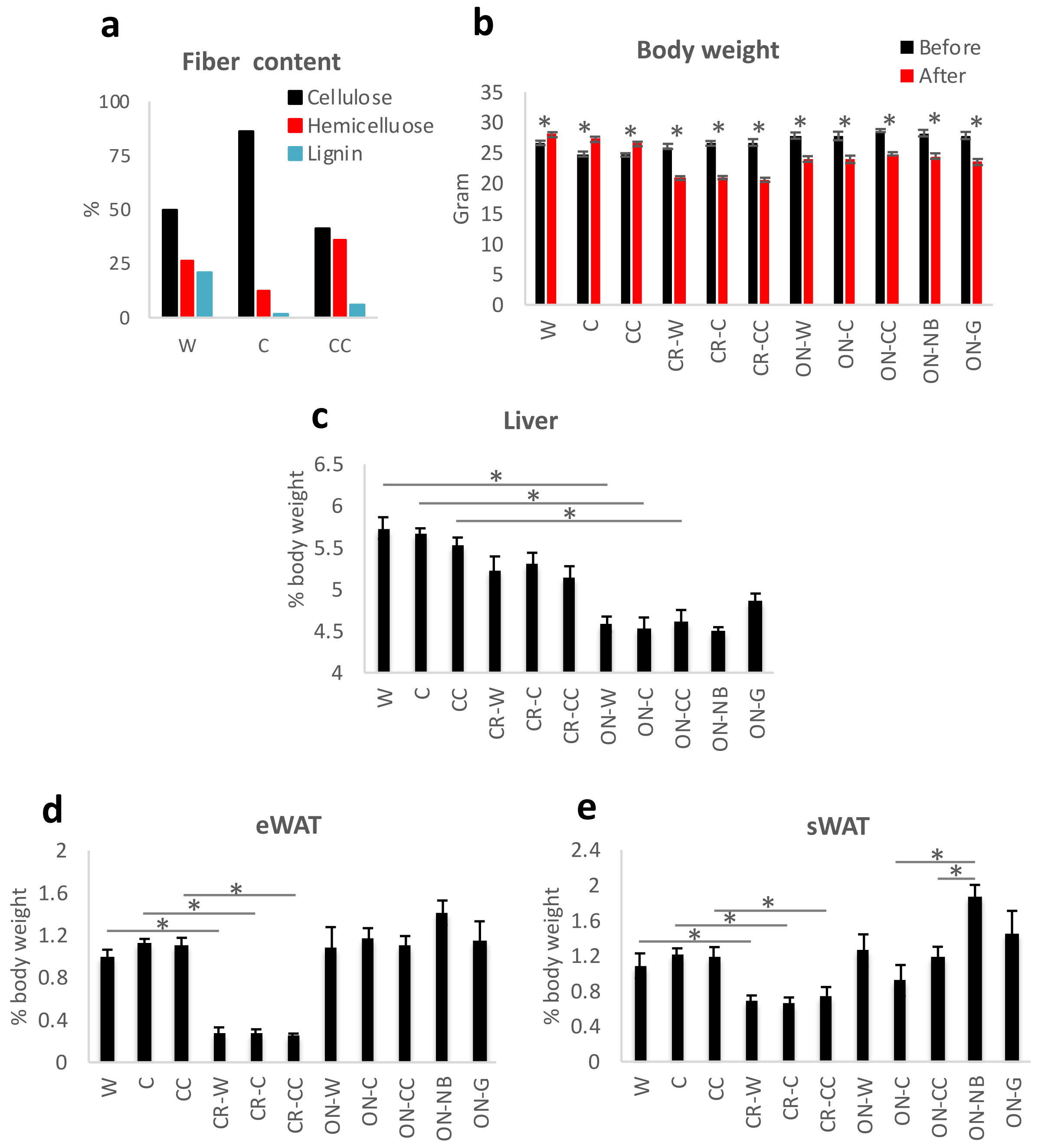

Supplementary figure S1: Fibre content of bedding and bedding's effect on body parameters. Bedding largely contains three main fibres in different ratios (a). Body weight was measured before and after the experiments (b) and two-tailed Student's t-tests were used to determine statistical significance; $p<0.05$. Liver (c), epididymal white adipose tissue (eWAT) (d) and subcutaneous white adipose tissue (sWAT) (e) weight was measured under *ad libitum* conditions, after 14 days of caloric restriction (CR) and after over-night (ON) fasting. Groups in panels c-e were compared using one-way ANOVA. Figures' bars stand for the mean of nine to ten biological replicates ±SEM.

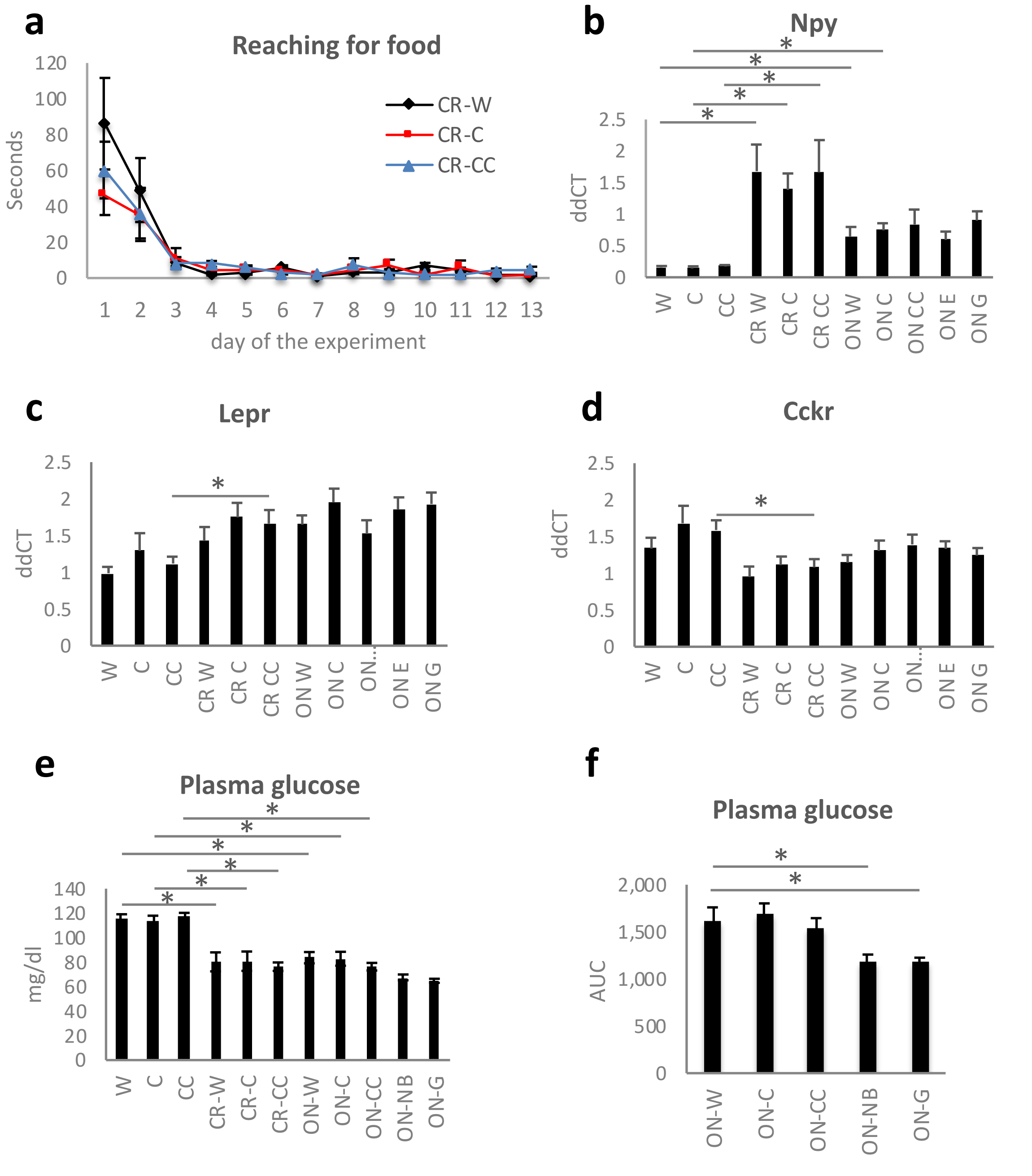

Supplementary figure S2: Hunger assessment and glucose tolerance. The daily amount of food was placed in the cage and the time was measured until mice initiated the meal (a). Gene expression of appetite regulating neuropeptide Y (Npy) (b), leptin-receptor (Lepr) (c), cholecystokinin receptor (Cckr) (d) were assayed in the hypothalamus by qRT-PCR. Fasting plasma glucose was measured in basic conditions (e) and over 2h following oral glucose bolus (f). Experimental groups were compared using one-way ANOVA. Error bars indicate ±SEM.

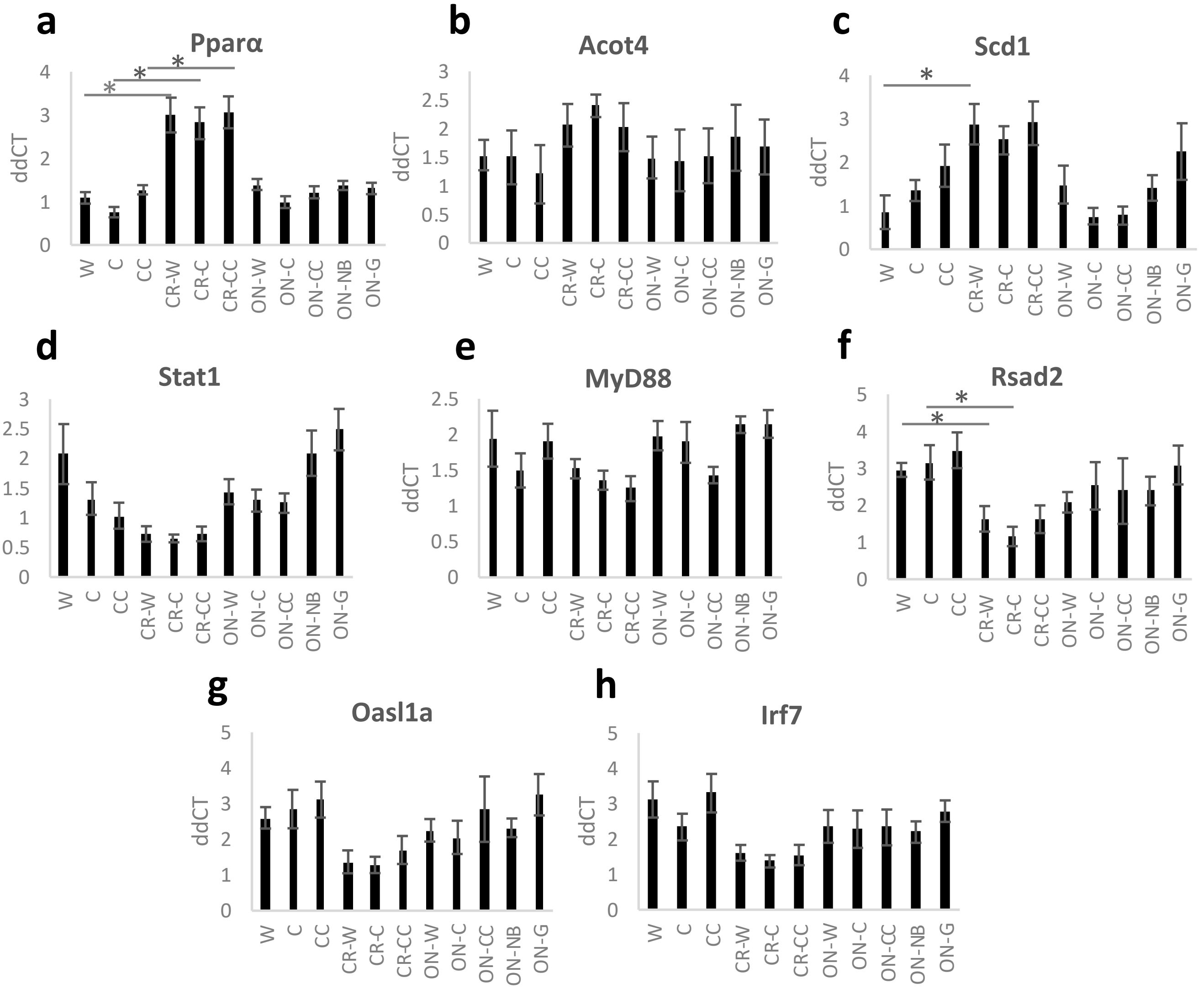

Supplementary figure S3: Gene expression of the intestinal mucosa. The gene expression of metabolic (a-c) and inflammatory (d-h) genes in the intestinal mucosa of mice were determined by qRT-PCR. Groups were compared using one-way ANOVA. * indicates statistical significance for Bonferroni post-hoc test. Error bars stand for ±SEM.

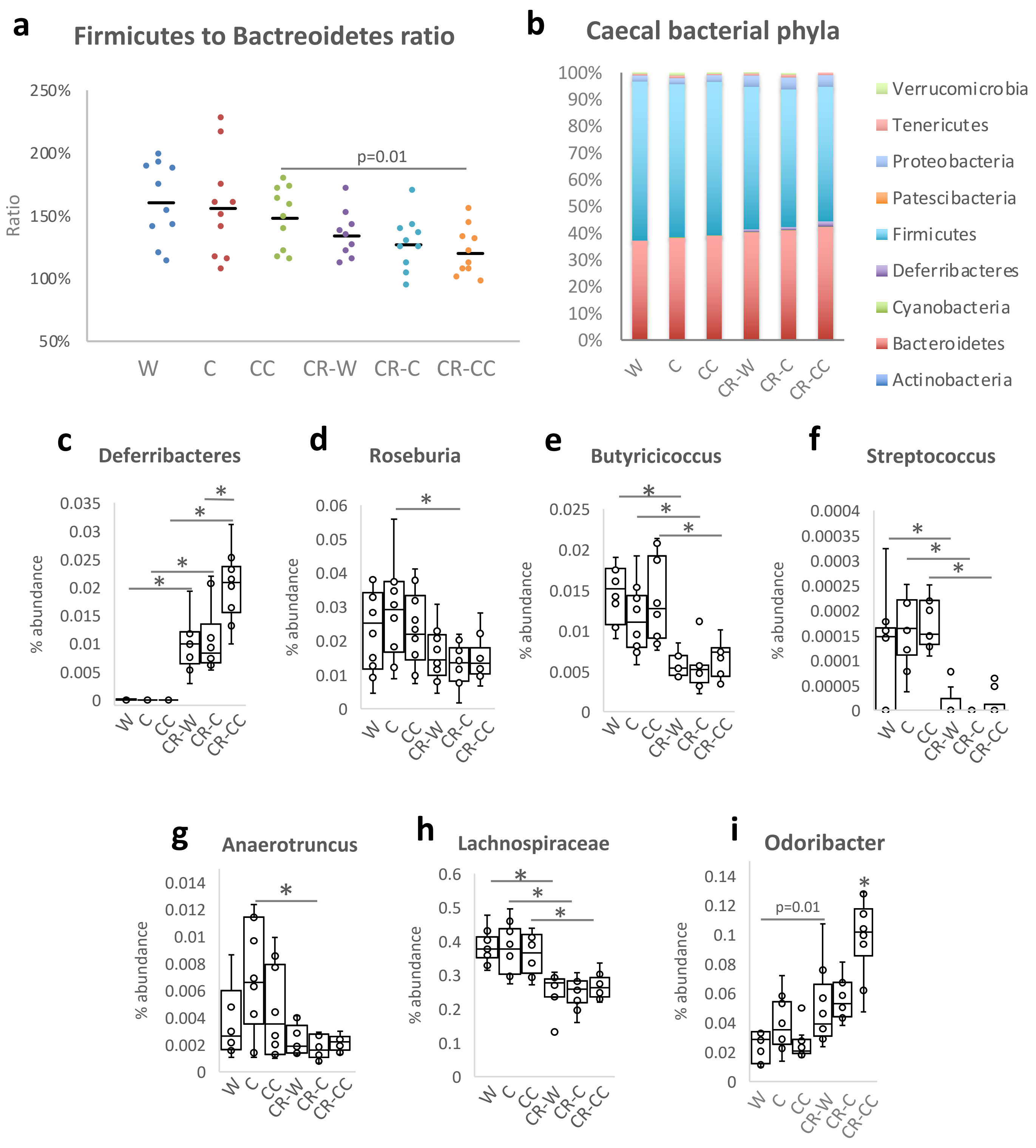

Supplementary figure S4: Effect of CR and bedding on the cecal microbiome. Changes in the *Firmicutes* to *Bacteroidetes* ratio (a), as well as, bacterial phyla are depicted (b). Boxplots of *Deferribacteres* (c), *Roseburia* (d), *Butyricoccus* (e), *Streptococcus* (f), *Anaerotruncus* (g), *Lachnospiraceae* (h), and *Odoribacter* (i) are shown. Groups were compared using one-way ANOVA. * indicates statistical significance following Bonferroni post-hoc test. In the panels a, c-i each circle represents one of nine to ten biological replicates.

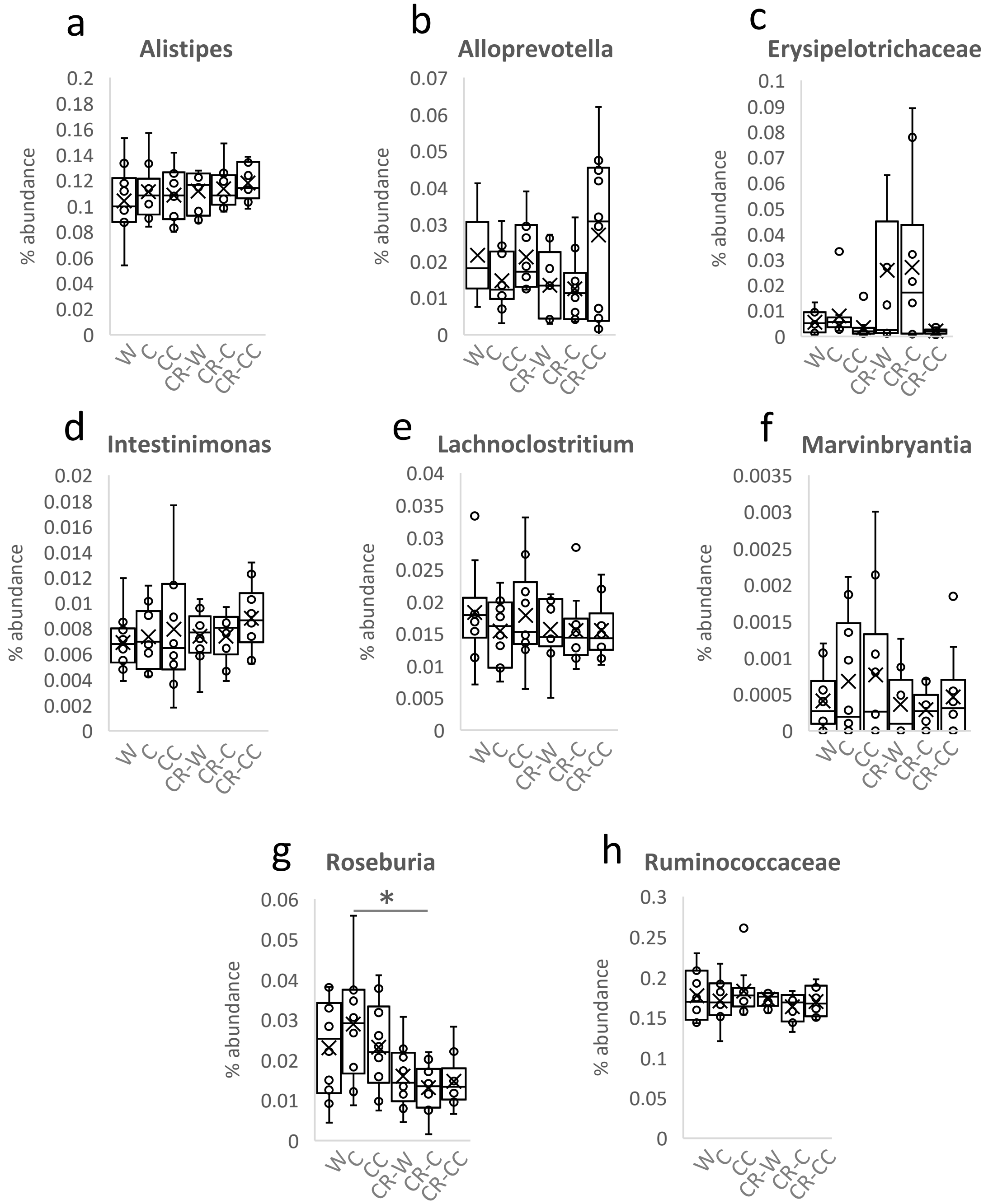

Supplementary figure S5: Effect of CR in the caecum content. Boxplots represent abundance of *Alistipes* (a), *Alloprevotella* (b), *Erysipelatichaceae* (c) *Intestinimonas* (d), *Lachnoclostritium* (e), *Marvinbryantia* (f), *Roseburia* (g), and *Ruminococcaceae* (h). Groups were compared using one-way ANOVA. * indicates statistical significance following Bonferroni post-hoc test. Each circle represents one of nine to ten biological replicates.

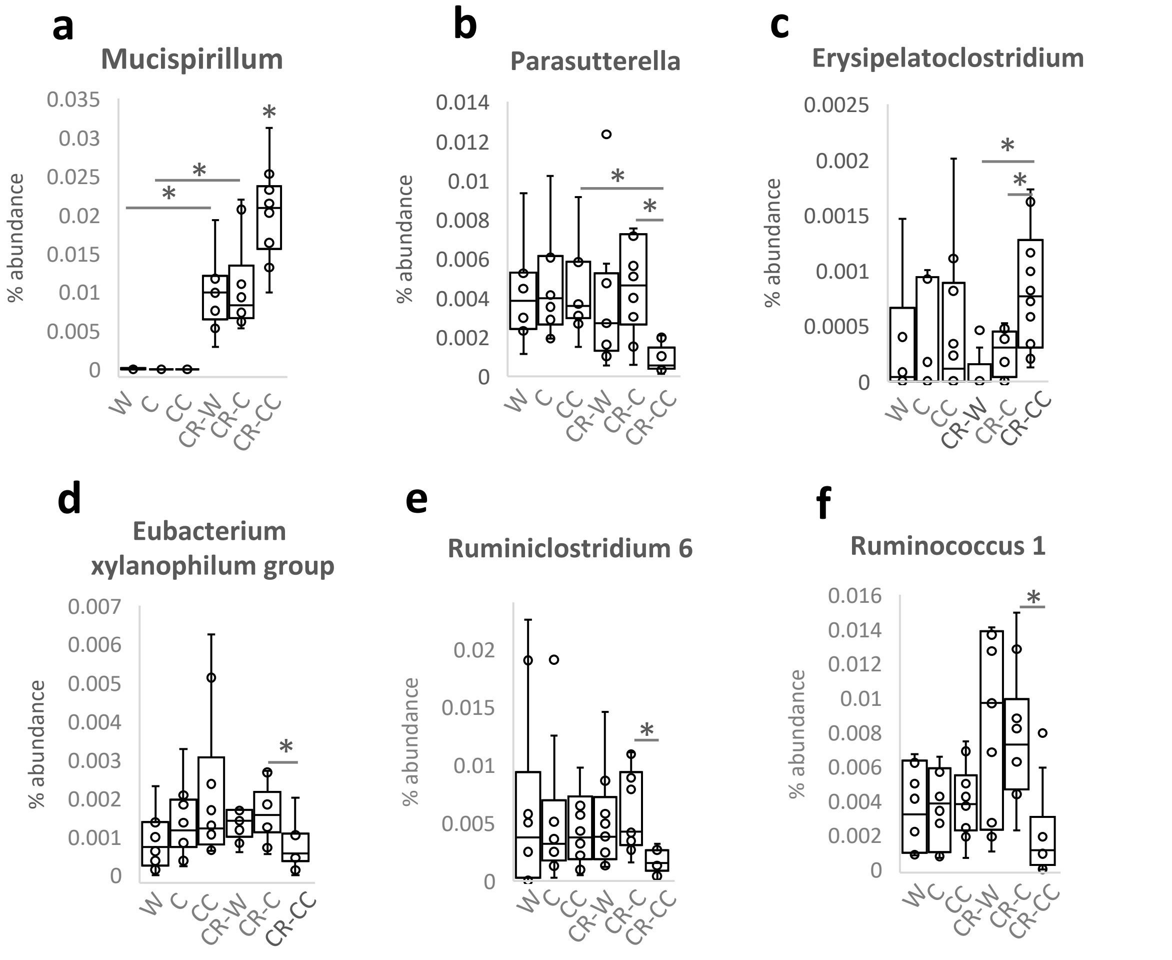

Supplementary figure S6: Effect of CR and bedding in the caecum content. Boxplots represent abundance of *Mucispirillum* (a), *Parasutterella* (b), *Erysipelatoclostridium* (c), *Eubacterium xylanophilum group* (d), *Ruminiclostridium 6* (e) and *Ruminococcus 1* (f). Groups were compared using one-way ANOVA. * indicates statistical significance following Bonferroni post-hoc test. Each circle represents one of nine to ten biological replicates.

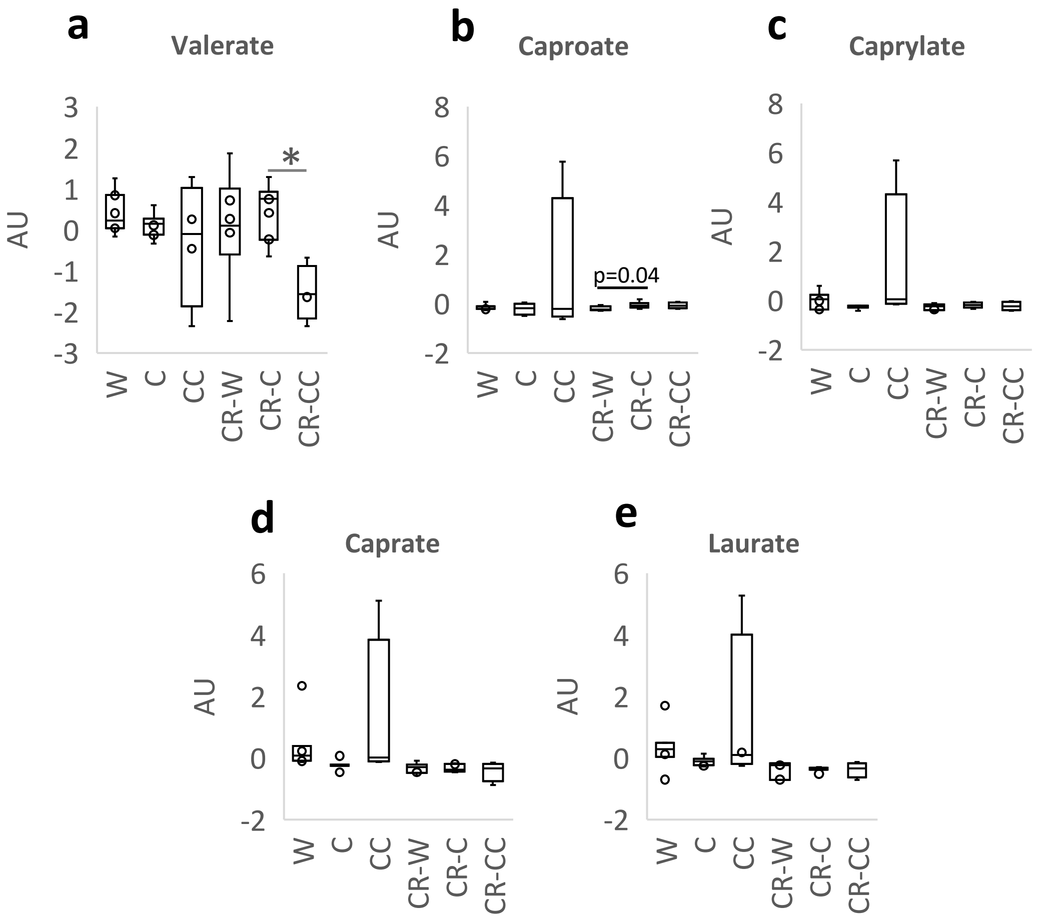

Supplementary figure S7: Cecal medium chain fatty acids levels. Z-Scored metabolites figures show the relative deviation from the groups mean value (0) for valerate (a), caproate (b), caprylate (c), caprate (d), and laurate (e) in the mice cecum content. Groups were compared using one-way ANOVA. * indicates statistical significance following Bonferroni post-hoc test. Each circle represents one nine to ten biological replicates.
