# Supplementary material for: Cage bedding modifies metabolic and gut microbiota profiles in mouse studies applying dietary restriction
Source: Sci Rep. 2020 Nov 30;10:20835. doi: 10.1038/s41598-020-77831-3 (PMC7705694; doi:10.1038/s41598-020-77831-3)
Supplement: Supplementary file 2 — Supplementary Information 2. [file 41598_2020_77831_MOESM2_ESM.pdf]

Supplementary Figures

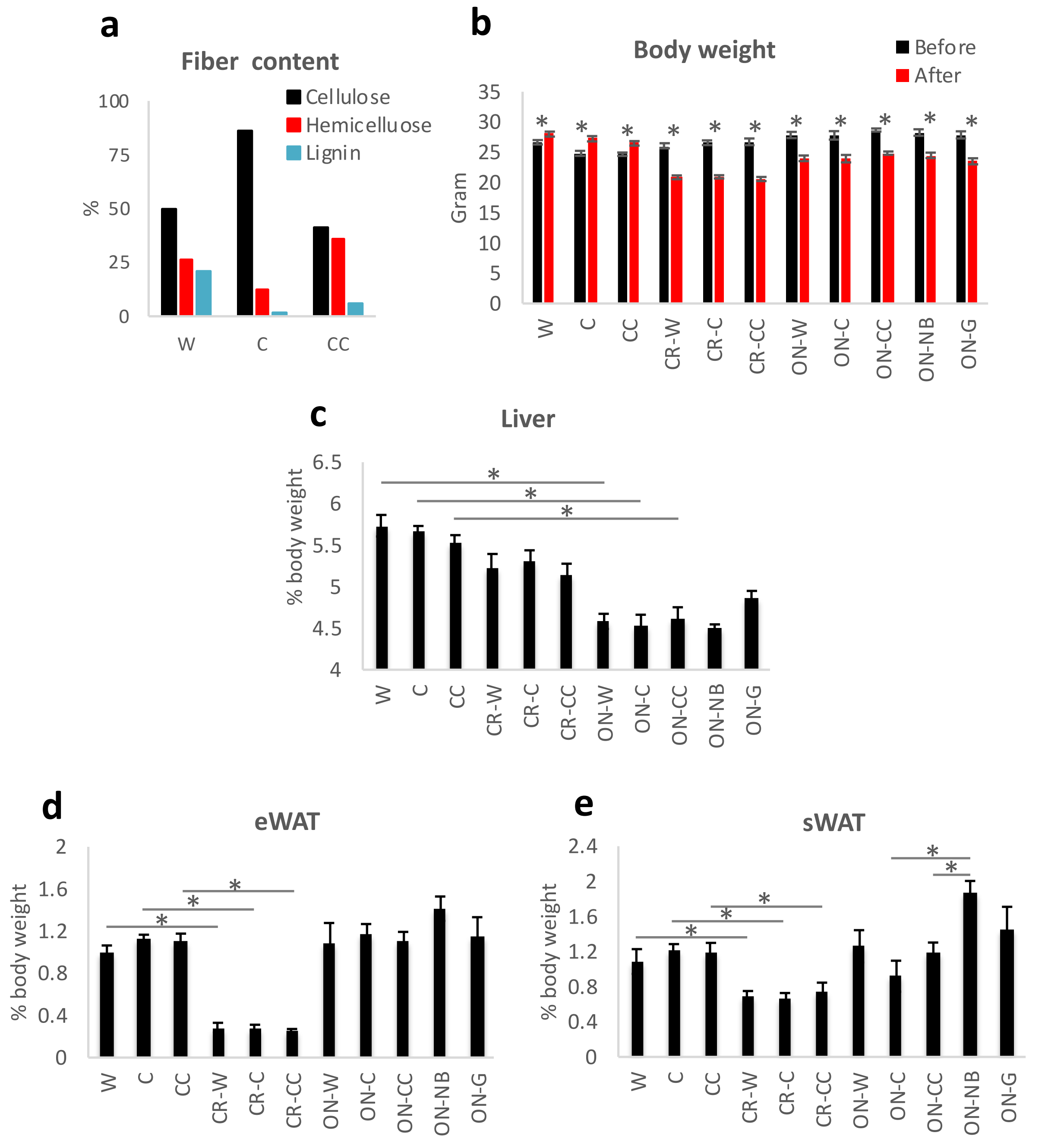

Supplementary figure S1: Fibre content of bedding and bedding's effect on body parameters. Bedding largely contains three main fibres in different ratios (a). Body weight was measured before and after the experiments (b) and two-tailed Student's t-tests were used to determine statistical significance; $p<0.05$. Liver (c), epididymal white adipose tissue (eWAT) (d) and subcutaneous white adipose tissue (sWAT) (e) weight was measured under *ad libitum* conditions, after 14 days of caloric restriction (CR) and after over-night (ON) fasting. Groups in panels c-e were compared using one-way ANOVA. Figures' bars stand for the mean of nine to ten biological replicates ±SEM.

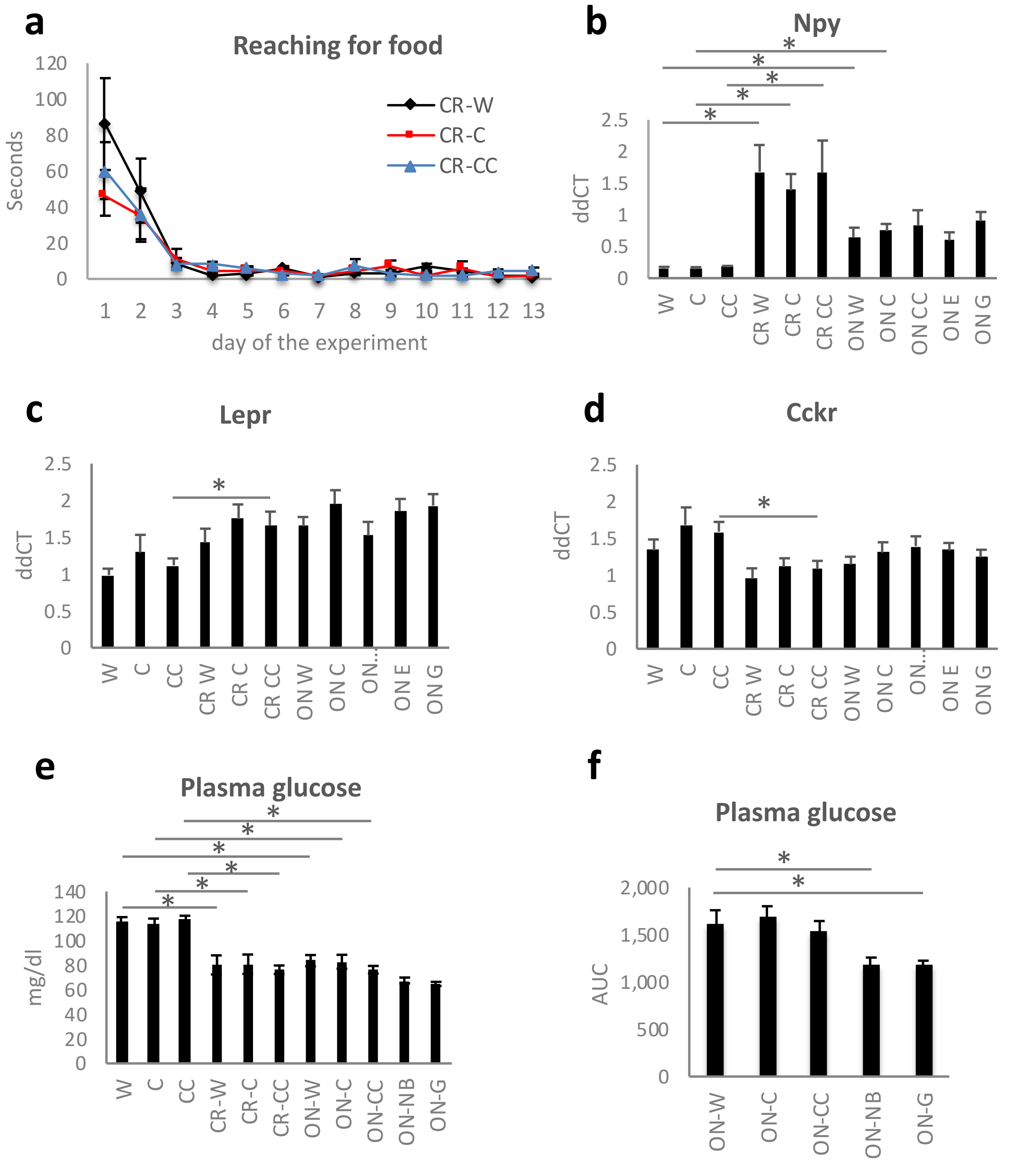

Supplementary figure S2: Hunger assessment and glucose tolerance. The daily amount of food was placed in the cage and the time was measured until mice initiated the meal (a). Gene expression of appetite regulating neuropeptide Y (Npy) (b), leptin-receptor (Lepr) (c), cholecystokinin receptor (Cckr) (d) were assayed in the hypothalamus by qRT-PCR. Fasting plasma glucose was measured in basic conditions (e) and over 2h following oral glucose bolus (f). Experimental groups were compared using one-way ANOVA. Error bars indicate ±SEM.

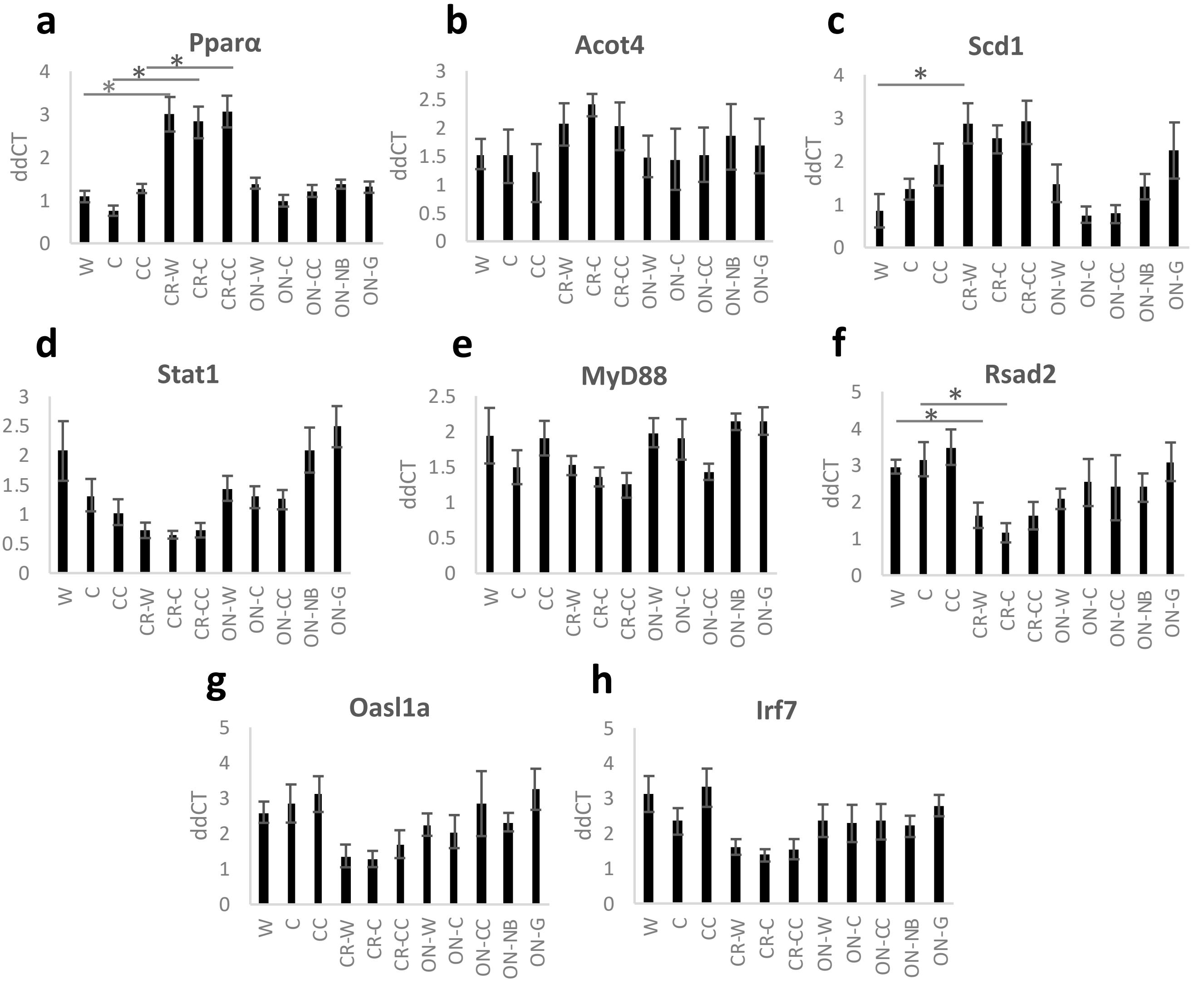

Supplementary figure S3: Gene expression of the intestinal mucosa. The gene expression of metabolic (a-c) and inflammatory (d-h) genes in the intestinal mucosa of mice were determined by qRT-PCR. Groups were compared using one-way ANOVA. * indicates statistical significance for Bonferroni post-hoc test. Error bars stand for ±SEM.

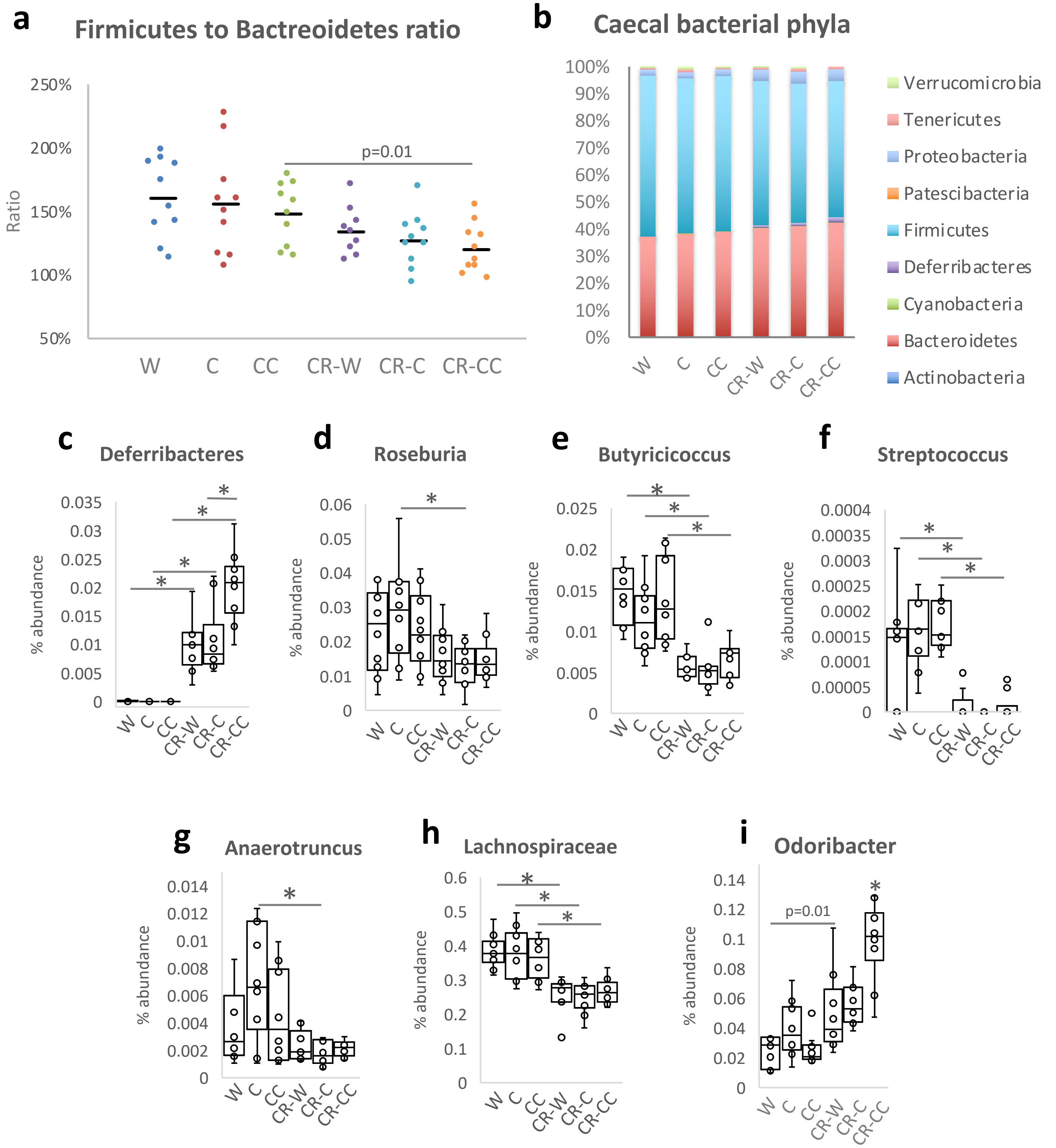

Supplementary figure S4: Effect of CR and bedding on the cecal microbiome. Changes in the *Firmicutes* to *Bacteroidetes* ratio (a), as well as, bacterial phyla are depicted (b). Boxplots of *Deferribacteres* (c), *Roseburia* (d), *Butyricoccus* (e), *Streptococcus* (f), *Anaerotruncus* (g), *Lachnospiraceae* (h), and *Odoribacter* (i) are shown. Groups were compared using one-way ANOVA. * indicates statistical significance following Bonferroni post-hoc test. In the panels a, c-i each circle represents one of nine to ten biological replicates.

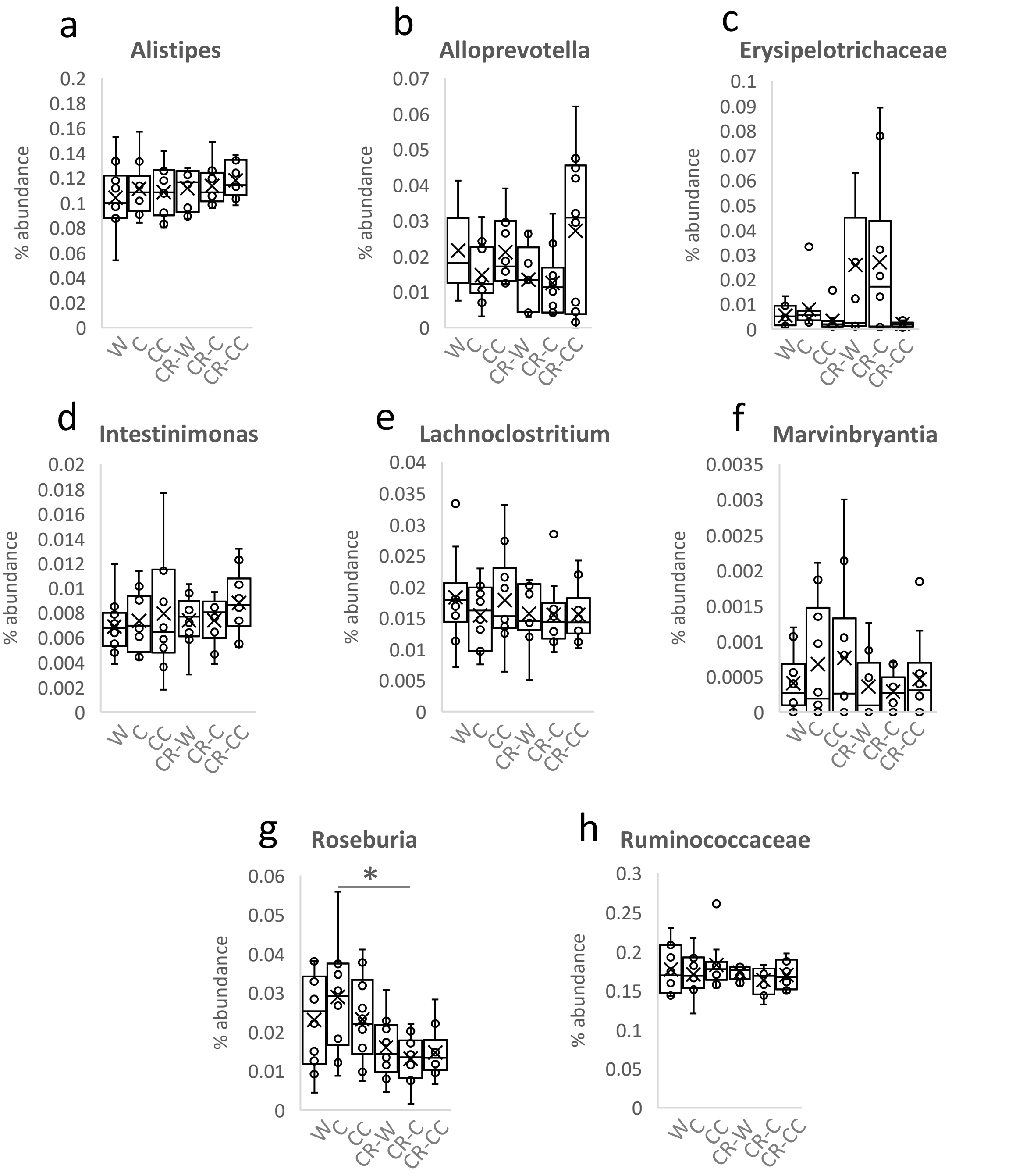

Supplementary figure S5: Effect of CR in the caecum content. Boxplots represent abundance of *Alistipes* (a), *Alloprevotella* (b), *Erysipelatichaceae* (c) *Intestinimonas* (d), *Lachnoclostritium* (e), *Marvinbryantia* (f), *Roseburia* (g), and *Ruminococcaceae* (h). Groups were compared using one-way ANOVA. * indicates statistical significance following Bonferroni post-hoc test. Each circle represents one of nine to ten biological replicates.

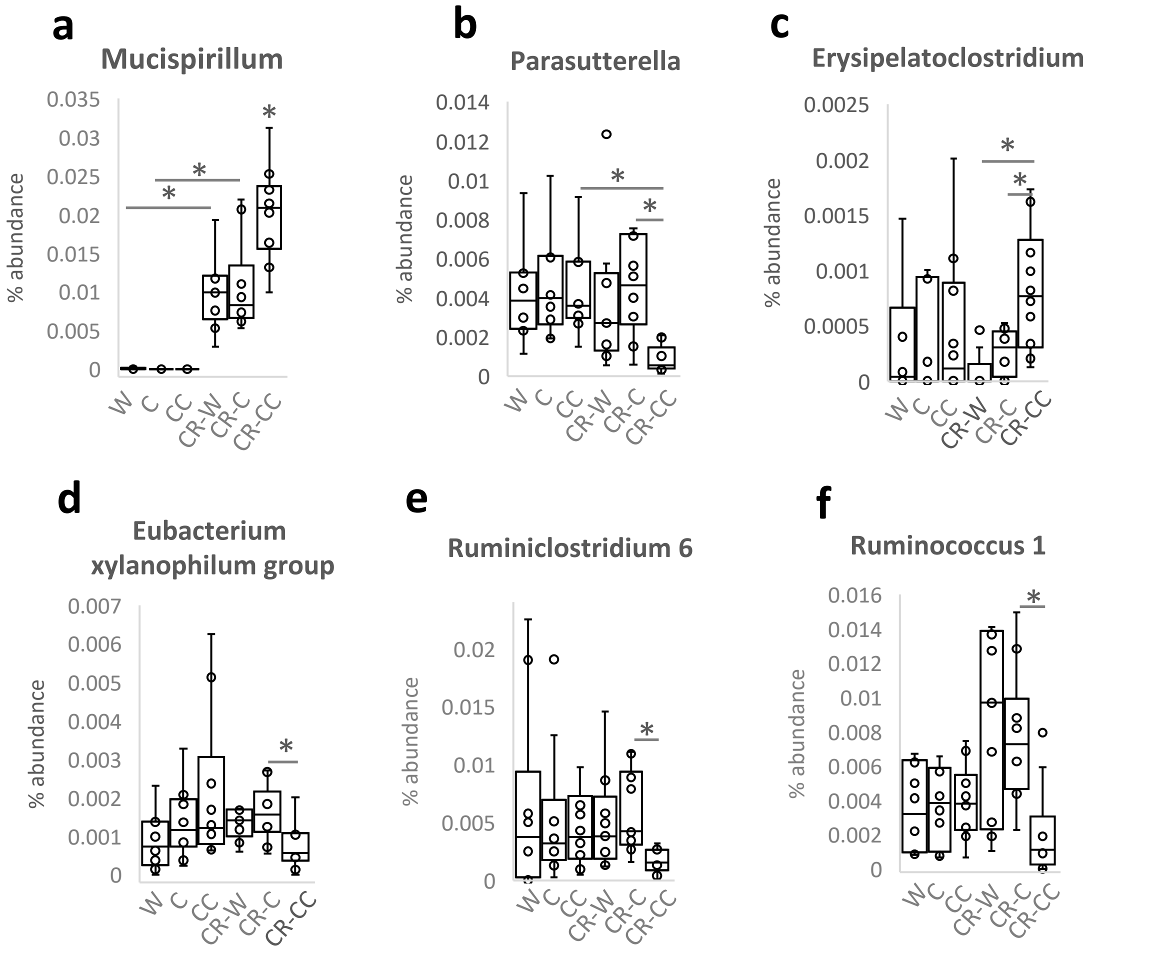

Supplementary figure S6: Effect of CR and bedding in the caecum content. Boxplots represent abundance of *Mucispirillum* (a), *Parasutterella* (b), *Erysipelatoclostridium* (c), *Eubacterium xylanophilum group* (d), *Ruminiclostridium 6* (e) and *Ruminococcus 1* (f). Groups were compared using one-way ANOVA. * indicates statistical significance following Bonferroni post-hoc test. Each circle represents one of nine to ten biological replicates.

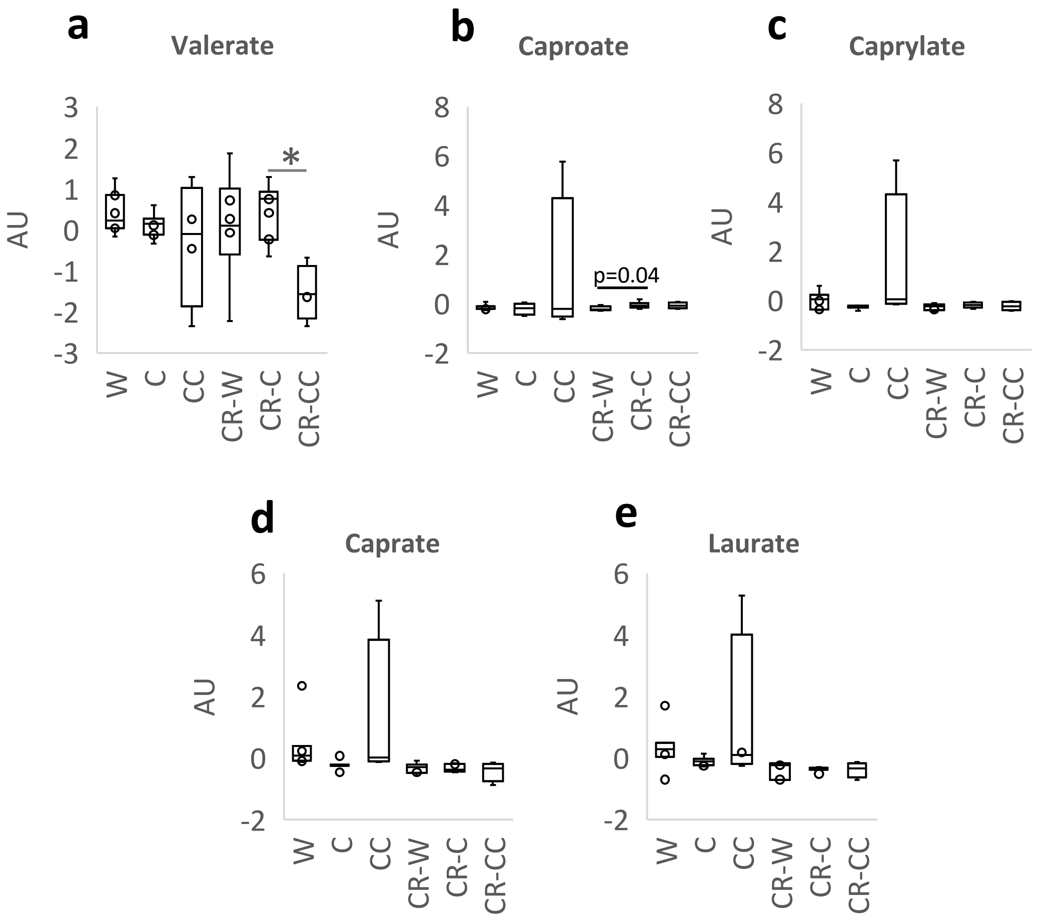

Supplementary figure S7: Cecal medium chain fatty acids levels. Z-Scored metabolites figures show the relative deviation from the groups mean value (0) for valerate (a), caproate (b), caprylate (c), caprate (d), and laurate (e) in the mice cecum content. Groups were compared using one-way ANOVA. * indicates statistical significance following Bonferroni post-hoc test. Each bar represents nine to ten biological replicates.
